# Identical Substitutions in Magnesium Chelatase Paralogs Result in Chlorophyll-Deficient Soybean Mutants

**DOI:** 10.1534/g3.114.015255

**Published:** 2014-12-01

**Authors:** Benjamin W. Campbell, Dhananjay Mani, Shaun J. Curtin, Rebecca A. Slattery, Jean-Michel Michno, Donald R. Ort, Philip J. Schaus, Reid G. Palmer, James H. Orf, Robert M. Stupar

**Affiliations:** * Department of Agronomy and Plant Genetics, University of Minnesota, St. Paul, Minnesota 55108; † Department of Plant Biology, University of Illinois, Urbana, Illinois 61801; ‡ US Department of Agriculture/Agricultural Research Service, Global Change and Photosynthesis Research Unit, Urbana, Illinois 61801; § Department of Agronomy, Iowa State University, Ames, Iowa 50011

**Keywords:** soybean, photosynthesis, chlorophyll, paralog, duplication

## Abstract

The soybean [*Glycine max* (L.) Merr.] chlorophyll-deficient line MinnGold is a spontaneous mutant characterized by yellow foliage. Map-based cloning and transgenic complementation revealed that the mutant phenotype is caused by a nonsynonymous nucleotide substitution in the third exon of a Mg-chelatase subunit gene (ChlI1a) on chromosome 13. This gene was selected as a candidate for a different yellow foliage mutant, T219H (*Y11y11*), that had been previously mapped to chromosome 13. Although the phenotypes of MinnGold and T219H are clearly distinct, sequencing of ChlI1a in T219H identified a different nonsynonymous mutation in the third exon, only six base pairs from the MinnGold mutation. This information, along with previously published allelic tests, were used to identify and clone a third yellow foliage mutation, CD-5, which was previously mapped to chromosome 15. This mutation was identified in the ChlI1b gene, a paralog of ChlI1a. Sequencing of the ChlI1b allele in CD-5 identified a nonsynonymous substitution in the third exon that confers an identical amino acid change as the T219H substitution at ChlI1a. Protein sequence alignments of the two Mg-chelatase subunits indicated that the sites of amino acid modification in MinnGold, T219H, and CD-5 are highly conserved among photosynthetic species. These results suggest that amino acid alterations in this critical domain may create competitive inhibitory interactions between the mutant and wild-type ChlI1a and ChlI1b proteins.

The soybean genetics community has an extensive history of identifying and collecting spontaneous mutant lines (Bernard 1975; Bernard *et al.* 1991). Differences in leaf chlorophyll content (SOY:0001858, SOY:0001859), specifically chlorophyll-deficient phenotypes, are one of the most extensively collected classes, with more than 20 different mutants encoded by the nuclear genome identified and phenotypically characterized (Palmer *et al.* 2004). These mutants exhibit a diverse range of phenotypes, including dominant and recessive alleles and minor, major, intermediate, and mosaic yellow foliage types. Universally, there is a loss of plant vigor associated with the yellow foliage phenotypes, with some mutations being more detrimental to overall fitness than others. Although some of these mutations have been genetically mapped (Palmer *et al.* 1989, 1990; Zou *et al.* 2003; Mahama and Palmer 2003; Kato and Palmer 2004; Zhang *et al.* 2011), to our knowledge no study has definitively identified the causative gene or nucleotide polymorphism underlying the phenotype.

The challenge of identifying gene functions in soybean is further complicated by the high rate of gene duplication in the paleopolyploid genome (Schmutz *et al.* 2010). Duplicate gene copies are thought to often mask the phenotypic consequences of mutating or silencing genes. The most recent whole-genome duplication in soybean was estimated to have occurred approximately 13 million years ago, and at least two copies are maintained for nearly 75% of the genes (Schmutz *et al.* 2010). Therefore, mutations derived *de novo* in these genes may be less likely to generate new phenotypes, as the duplicate copies may mask the loss of gene functions.

The recent development of genomic and mapping tools in the soybean community has facilitated map-based cloning efforts for specific traits in recent years (*e.g.*, Liu *et al.* 2012; Tian *et al.* 2010; reviewed by Stupar and Specht 2013). In this study, we have used modern genomic tools to rapidly map and fine-map a recently discovered spontaneous chlorophyll-deficient mutant line known as MinnGold. The reference soybean genome sequence (Schmutz *et al.* 2010) was used to identify a Mg-chelatase subunit ChlI1a homolog as a candidate gene. The ChlI Mg-chelatase subunit is involved in catalyzing the insertion of Mg^2+^ into the protoporphyrin IX to form the first committed step in the chlorophyll biosynthesis pathway. Sequencing and functional analyses revealed that a specific nucleotide substitution in the coding region of this gene is likely responsible for the chlorophyll-deficient phenotype of MinnGold.

Knowledge gained from the cloning of the ChlI Mg-chelatase gene was then used to identify the causal mutations underlying *y11* and CD-5, two chlorophyll-deficient alleles that were initially identified decades ago (Weber and Weiss 1959; Palmer *et al.* 1989). A distinct nucleotide substitution six base pairs from the MinnGold mutation was identified in *y11*, consistent with previous mapping of this locus (Mahama and Palmer 2003). Furthermore, allelism results of *y11* and CD-5 (Palmer *et al.* 1989) provided evidence for a Mg-chelatase paralog on chromosome 15 as a candidate gene for the CD-5 chlorophyll-deficient phenotype. Sequencing of the Mg-chelatase gene on chromosome 15 identified a nonsynonymous single-nucleotide polymorphism (SNP) change also in the third exon. Interestingly, the positions and base changes of the *y11* and CD-5 nonsynonymous polymorphisms result in identical amino acid substitutions.

## Materials and Methods

### Discovery and phenotype analysis of the MinnGold mutant

In 2008, several chlorophyll-deficient plants were identified in a segregating row at a University of Minnesota winter nursery in Chile. These mutants were observed in a F_3_ population derived from a cross between M99-274166 × ‘MN0091’. ‘MN0091’ is a cultivar release from the University of Minnesota Soybean Breeding Program, and M99-274166 is a selection from the cross PI 548379 × (Mandarin Ottawa × NK S19-90). Each of the chlorophyll-deficient plants was threshed and maintained as separate lines, and one of these lines was given the name MinnGold. To determine chlorophyll content, tissue was collected from the second true leaves at the V5 leaf stage of several MinnGold and ‘Williams 82’ plants and frozen in foil packets shortly after collection. Five 1-cm leaf disks were taken from frozen tissue of each cultivar. Disks were ground in cold methanol and centrifuged according to Porra *et al.* (1989), and absorbance was determined following the methods described by Lichtenthaler (1987) and Wellburn (1994).

### Genetic mapping of chlorophyll deficiency in MinnGold

The chlorophyll-deficient mutant line MinnGold was crossed to soybean accessions ‘Archer’ and ‘Minsoy’ to develop populations for genetic mapping experiments. The F_2_ progeny were grown in standard greenhouse conditions and visually phenotyped for relative chlorophyll content. Bulk segregant analysis (BSA) (Michelmore *et al.* 1991) was conducted on F_2_ individuals using the Illumina soybean 1536 SNP chip for genotyping (Hyten *et al.* 2008, 2009). A DNeasy Plant Mini Kit (QIAGEN) was used for the DNA extraction from the fresh bulk leaf tissue and for all subsequent DNA extractions from fresh leaf tissue unless otherwise stated. The wild-type and mutant bulks for both populations were composed of leaf tissue from 20 green and 20 yellow plants, respectively. BSA results from the two populations were combined to identify the approximate chromosomal position of the causative mutation.

Seeds were harvested from each F_2_ individual to generate F_2:3_ families, which were subsequently planted in the greenhouse. Leaf tissues were collected from one individual in each F_2:3_ family, and DNA was extracted using a BioSprint 96 DNA Plant Kit (QIAGEN) following the manufacturer’s protocol. To narrow the mapped interval, the F_2:3_ individuals were genotyped at the University of Minnesota Genomics Center (UMGC) core facility using a custom panel of 30 SNP assays (Supporting Information, Table S1) on a Sequenom MassARRAY genotyping platform. Five F_3_ individuals were identified as heterozygous in the mapped interval, and these individuals were harvested and advanced to generate segregating F_3:4_ families. F_3:4_ families were grown in the greenhouse and leaf tissue was collected from each individual for DNA extractions, as described above. The F_3:4_ individuals were subjected to an additional round of fine-mapping on a second custom Sequenom MassARRAY genotyping platform (70 SNPs; Table S2) to further narrow the mapped interval. The Sequenom MassARRAY SNP panels for both rounds of fine mapping were designed based on assays from the SoySNP50K set (Song *et al.* 2013). The fine-mapped interval included gene model Glyma13g30560 (renamed as Glyma.13G232500 in the Glyma.Wm82.a2.v1 annotation), a putative Mg-chelatase subunit ChlI1a.

### Identification of Glyma13g30560 as a candidate for *y11*

Mahama and Palmer (2003) conducted linkage mapping of multiple phenotypic traits on chromosome 13 and established the locus order: *y11*, a semidominant yellow foliage mutation; *w1*, a white flower mutation; and *y23*, a yellow foliage mutation. Their mapping data suggested that *w1* and *y23* were more closely linked and that *y11* was more distantly linked to *w1*. The combination of the mapping data from Mahama and Palmer (2003) and Zabala and Vodkin (2007) indicate that the *w1* locus and *y23* are on the opposite chromosome arm as the F_3:4_ fine-mapped interval, indicating that the *y11* and MinnGold mutations are likely on the same chromosome arm. Furthermore, the mapping results by Mahama and Palmer (2003) did not rule out the possibility that the mutation in MinnGold could be allelic to *y11*.

### Sequencing of Glyma13g30560 and Glyma15g08680 in mutant and wild-type lines

The candidate gene for the MinnGold phenotype, Glyma13g30560, was sequenced from a homozygous mutant and a homozygous wild-type plant to identify the presence of polymorphisms. Homozygous individuals were identified as individuals of families not segregating for the foliage phenotype. The RNA was extracted from fresh leaf tissue using a QIAGEN RNAeasy kit following the manufacturer’s protocol. The RNA was reverse transcribed using SuperScript III reverse transcriptase (Invitrogen) following the manufacturer’s protocol. The cDNA sequence for Glyma13g30560 was amplified with polymerase chain reaction (PCR) from the plants using primers 5′ TACAGTCTGTCTTCTCTTCTCTTCTCCGG 3′ and 5′ GAATACAAACCGTGTTACATCTATGATCC 3′. The PCR amplicons from this reaction, and subsequent PCRs unless otherwise stated, were purified using a QIAquick PCR Purification Kit (QIAGEN) following the manufacturer’s protocol and sequenced using the Sanger sequencing method at the UMGC.

The candidate genes for *y11* and for CD-5, Glyma13g30560 and Glyma15g08680 (renamed as Glyma.15g080200 in the Glyma.Wm82.a2.v1 annotation), respectively, also were sequenced to identify the presence of polymorphisms. The DNA was extracted from fresh leaf tissue of two *Y11/Y11* and two *y11/y11* individuals. Glyma13g30560 was amplified with PCR from the four individuals using a set of PCRs with overlapping amplicons (Table S3). Several long PCRs with a unique primer outside of the gene were needed to amplify the internal sequence of Glyma13g30560, which is highly conserved between the four soybean homologs. To obtain quality sequences for the internal sections of the gene, these long PCR amplicons were gel extracted using a QIAquick Gel Extraction Kit (QIAGEN) following the manufacturer’s protocol and were used as the template for a second round of PCR using primers that anneal to the internal sequences of Glyma13g30560. This method prevented the contamination of the Glyma13g30560 sequences with sequences from the other soybean homologs. All PCRs were conducted using the proof reading KOD DNA polymerase enzyme (Novagen). The resulting PCR amplicons were then purified. The same steps of PCR amplification, gel extraction, a second round of PCR, and PCR purification were also used to sequence Glyma15g08680 in two homozygous wild-type and two CD-5/CD-5 individuals (primers in Table S4). The final PCR amplicons were then sequenced. Sequences were aligned using Mega 5 software (Tamura *et al.* 2011).

To test for the occurrence of the *y11* and MinnGold candidate SNPs in a diverse set of the soybean germplasm, the portion of the third exon of Glyma13g30560 containing the candidate SNPs was PCR amplified from 29 diverse parent lines (McHale *et al.* 2012) using primers 5′GGCCAGGCCTTTGCATTTTG 3′ and 5′ACTCAGCACACACCTTGGAG 3′. To test for the occurrence of the CD-5 candidate SNP in the soybean germplasm, the portion of the third exon of Glyma15g08680 containing the SNP was also PCR amplified from these 29 diverse parent lines using primers 5′GGCTAGGCCTTTGTGTTTGA 3′ and 5′AACGGGAAATGCTGATTGAG 3′. The resulting PCR amplicons were then purified and sequenced. Sequences were aligned using Mega 5 software (Tamura *et al.* 2011).

### CAPS assays for *y11* and CD-5

The candidate *y11* SNP change creates a *Sac*II digestion site that can be easily screened by a cleaved amplified polymorphic sequences (CAPS) assay. Thus a CAPS assay was used to genotype additional individuals segregating for *y11* to test if the candidate SNP cosegregated with the chlorophyll deficient phenotype. Genomic DNA was extracted from fresh leaf tissue from 19 segregating progeny derived from a single heterozygous (*Y11/y11*) plant. The portion of the third exon containing the candidate SNP was PCR amplified using primers 5′GGCCAGGCCTTTGCATTTTG 3′ and 5′ACTCAGCACACACCTTGGAG 3′. The PCR was then digested with *Sac*II (New England BioLabs) following the manufacturer’s protocol and run on a 1% agarose gel. The *Sac*II digestion cuts the mutant strand forming band sizes of 237 bp and 691 bp.

The same techniques were also used to conduct a CAPS assay for CD-5. In summary, the candidate CD-5 SNP change removes an AlwNI digestion site that can be easily screened by a CAPS assay. As before, a CAPS assay was used to genotype 17 segregating individuals derived from a single heterozygous (wild-type*/*CD-5) plant to test for cosegregation of the candidate SNP with the chlorophyll deficient phenotype. Genomic DNA was extracted from fresh leaf tissue, and the portion of the third exon containing the candidate SNP was PCR amplified using primers 5′ GGCTAGGCCTTTGTGTTTGA 3′ and 5′AACGGGAAATGCTGATTGAG 3′. The PCR was then digested with AlwNI (New England BioLabs) following the manufacturer’s protocol and run on a 1% agarose gel. The AlwNI digestion cuts the wild-type strand forming band sizes of 695 bp and 803 bp.

### Transformation and transgene analysis

MinnGold was transformed with the wild-type allele of Glyma13g30560 for complementation analysis. Glyma13g30560 was amplified with PCR from wild-type cultivar ‘Williams 82’ using a proof reading KOD DNA polymerase enzyme (Novagen) and the following forward and reverse primers: 5′ GCTCACATGCGCGGCCGCTGGCACCCACTAACATTTCC 3′ and 5′ GCTCTCATGCCCTGCAGGCGAGGAAAGAGAATGGATGG 3′, respectively. The PCR amplified 4416 bp, spanning the region 777 bp upstream of the 5′ untranslated region (UTR) to 965 bp downstream of the 3′ UTR. The forward and reverse primers were designed with *Not*I and *Sbf*I restriction sites, respectively. The PCR fragment was gel extracted using the QIAquick Gel Extraction Kit (QIAGEN) following the manufacturer’s protocol, and the fragment was cloned into the pCR-Blunt II-TOPO vector (Invitrogen) following the manufacturer’s protocol. Insertion of the clone into the pCR-Blunt II-TOPO vector was confirmed using an internal primer 5′ GCACCTTCAAGCTTCGCTTT 3′ and M13 Forward and M13 Reverse primers 5′ GTAAAACGACGGCCAG 3′ and 5′CAGGAAACAGCTATGA 3′, respectively. Next, the fragment was transferred from the pCR-Blunt II-TOPO vector to the binary vector pNB96 (Fusaro *et al.* 2006) using a restriction digest with *Not*I and SbfI followed by ligation. The resulting clone was sequenced at the UMGC. The construct was transformed into MinnGold following the whole plant transformation and herbicide selection protocol described by Curtin *et al.* (2011). DNA was extracted from fresh T_0_ leaf tissue, and the whole plant transformation was confirmed by Southern Blot analysis of both the MinnGold parent (negative control) and the transformed T_0_ MinnGold plant.

The green T_0_ plant was self-pollinated to produce segregating T_1_ progeny. To test whether the transgene cosegregated with the wild-type (green) phenotype, DNA was extracted from fresh T_1_ leaf tissue for PCR analysis. PCR primers 5′ AAGATGTTTCTCCCCCATCC 3′ and 5′ CGTCTTGATGAGACCTGCTG3′ were used to screen for the presence of the transgene in seventeen of the segregating T_1_ progeny. The forward and reverse primers were designed to amplify a 127-bp fragment spanning from the end of the Glyam13g30560 genomic clone to the beginning of the CaMV35S promoter that precedes the BAR gene in the construct. Primers 5′ GAGCTATGAATTGCCTGATGG 3′ and 5′ CGTTTCATGAATTCCAGTAGC 3′ were used to amplify a 118-bp fragment of the soybean actin gene Glyma15g05570 as a positive PCR control.

### Analysis of sequence similarity between the soybean ChlI subunits

To compare the similarity of the four soybean ChlI subunits, a unweighted pair group method with arithmetic mean neighbor joining tree was constructed in MEGA5 by the unweighted pair group method with arithmetic mean method (Sneath and Sokal 1973) using amino acid sequences. The Poisson correction method was used to compute the evolutionary distances, which were calculated in units of the number of amino acid substitutions per site (Zuckerkandl and Pauling 1965). Positions containing gaps and missing data were eliminated, and a total of 415 positions were used in the final dataset. MEGA5 was used to conduct the evolutionary analyses (Tamura *et al.* 2011).

## Results

### Identification, mapping, and fine-mapping of the MinnGold mutation

**Figure 1:**
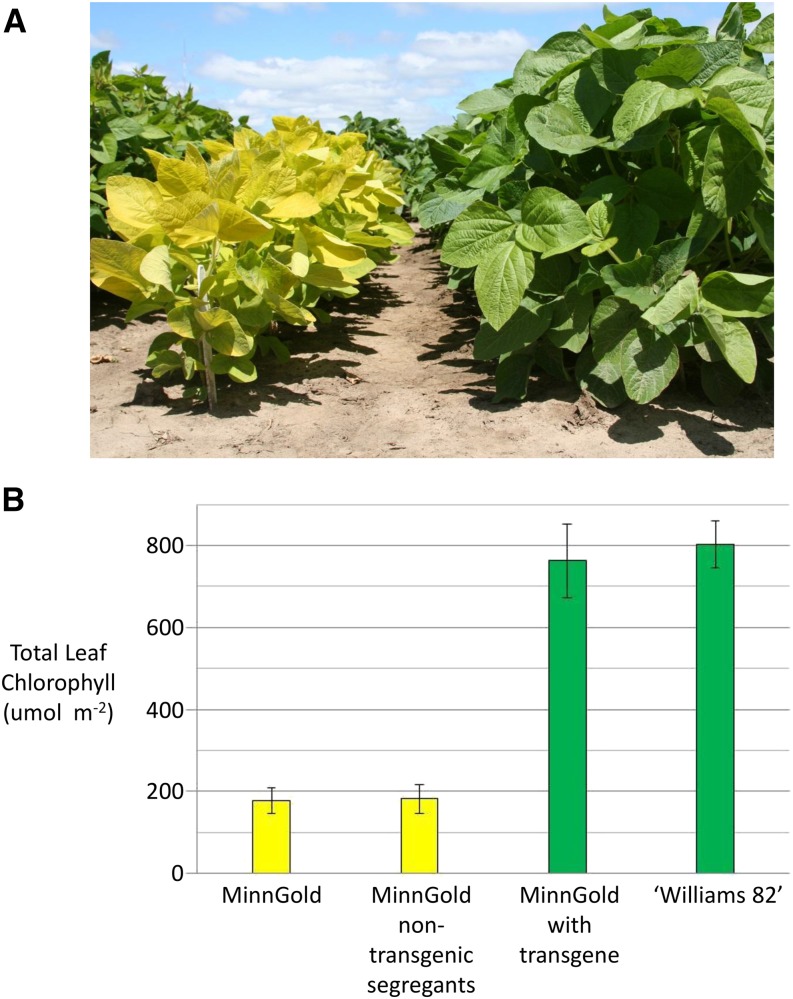
Phenotypic evaluation of chlorophyll deficiency in the MinnGold mutant. (A) Visual comparison of the MinnGold mutant (left) *vs.* the wild-type cultivar ‘Williams 82’ (right). (B) Total leaf tissue chlorophyll levels in the MinnGold, MinnGold non-transgenic segregants, Transgenic MinnGold T_1_, and ‘Williams 82’.

The spontaneous, nonlethal yellow foliage mutant (Figure 1) was first observed in a segregating F_3_ population derived from a cross between M99-274166 × ‘MN0091’. The F_3_ population was advanced by modified single seed descent and exhibited a segregation ratio of 157 green (wild-type) plants to 50 yellow (mutant) individuals. The ratio of 157:50 for the wild-type:chlorophyll-deficient phenotype is consistent with a single-locus, recessive mutation (χ^2^ test, *P* = 0.78). The mutant phenotype appears more vigorous than previously identified chlorophyll-deficient soybean mutants. Measurement of leaf chlorophyll levels from tissue collected from the second true leaves at the V5 leaf stage show that this mutant displayed a dramatic reduction of both chlorophyll a and chlorophyll b relative to wild-type soybean plants (Figure 1). This mutant was given the name MinnGold because of its bright yellow leaf coloration during early foliar development.

**Figure 2:**
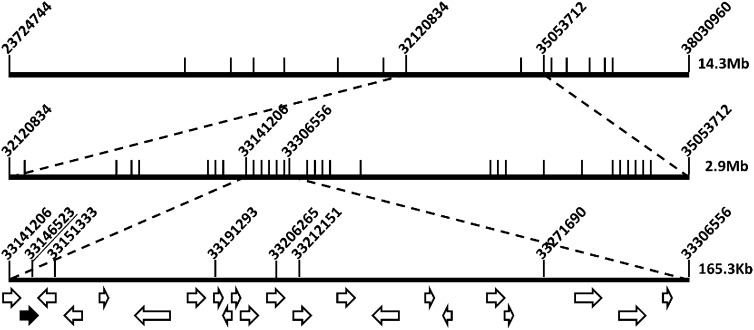
Fine mapping the MinnGold locus. Bulk segregant analysis mapping (top panel) followed by two rounds of fine mapping (middle and lower panels) narrowed the region containing the candidate gene to 165.3 kb on chromosome 13. The interval contains 22 genes indicated as arrows. The candidate gene is indicated with a black-filled arrow. The candidate SNP position located within the third exon of Glyma13g30560 at 33146523 is underlined. Marker positions are labeled based on the soybean genome assembly version Glyma.Wm82.a1 (Gmax1.01).

MinnGold was crossed to two soybean accessions, ‘Archer’ and ‘Minsoy’, to develop mapping populations. The F_2_ progeny from both populations exhibited segregation ratios of 3:1 for the wild-type:chlorophyll-deficient phenotype. The yellow foliage phenotype was observed in 79 of 311 plants (χ^2^ test, *P* = 0.87) derived from the MinnGold × ‘Archer’ cross. The yellow foliage phenotype was observed in 48 of 186 plants (χ^2^ test, *P* = 0.80) derived from the MinnGold × ‘Minsoy’ cross. These results further indicate that the phenotype is caused by a recessive allele at a single, nuclear locus. BSA (Michelmore *et al.* 1991) was conducted on wild-type and mutant F_2_ bulks from both populations. The BSA populations were genotyped using the Golden Gate 1536 SNP chip assay (Hyten *et al.* 2009) to narrow the region of interest to a 14.3 Mb interval on chromosome 13 (Figure 2).

Genotyping of 372 F_3_ individuals and parental lines was subsequently performed using a customized panel of 30 SNP markers (Table S1) within this chromosome 13 region, reducing the interval to 2.9 Mb. This interval was flanked by markers at positions 32,120,834 and 35,053,712 based on the soybean genome assembly version Glyma.Wm82.a1 (Figure 2). Five green F_3_ individuals that were genotyped as potentially heterozygous at or within either flanking marker were also found to segregate for the mutant phenotype in the F_3:4_ generation. In total, 159 F_3:4_ individuals from these families, along with a subset of F_3_ individuals and parental line controls, were phenotyped and genotyped with a custom panel of 70 SNP markers (Table S2). After this round of mapping, the mutation was mapped to a 165.3-kb interval on chromosome 13, residing between positions 33,141,206 and 33,306,556 (Figure 2).

### Identification of the candidate gene

According to the soybean genome assembly version 1.1 (Schmutz *et al.* 2010), the 163.5-kb interval contains 22 annotated genes. This list (Table S5) includes Glyma13g30560, a gene encoding a putative Mg-chelatase subunit ChlI1a. This gene was a promising candidate for the mutant phenotype since Mg-chelatase is involved with a major step in chlorophyll biosynthesis. Additionally, previous reports in thale cress (*Arabidopsis thaliana*) (Mochizuki *et al.* 2001; Rissler *et al.* 2002; Kobayashi *et al.* 2008; Huang and Li 2009), rice (*Oryza sativa*) (Zhang *et al.* 2006; Jung *et al.* 2003), barley (*Hordeum vulgare*) (Hansson *et al.* 1999; Hansson *et al.* 2002; Jensen *et al.* 1996), maize (*Zea mays*) (Sawers *et al.* 2006), and tobacco (*Nicotiana tabacum*) (Nguyen 1995) have associated mutations in Mg-chelatase subunits with chlorophyll deficient phenotypes.

**Figure 3:**
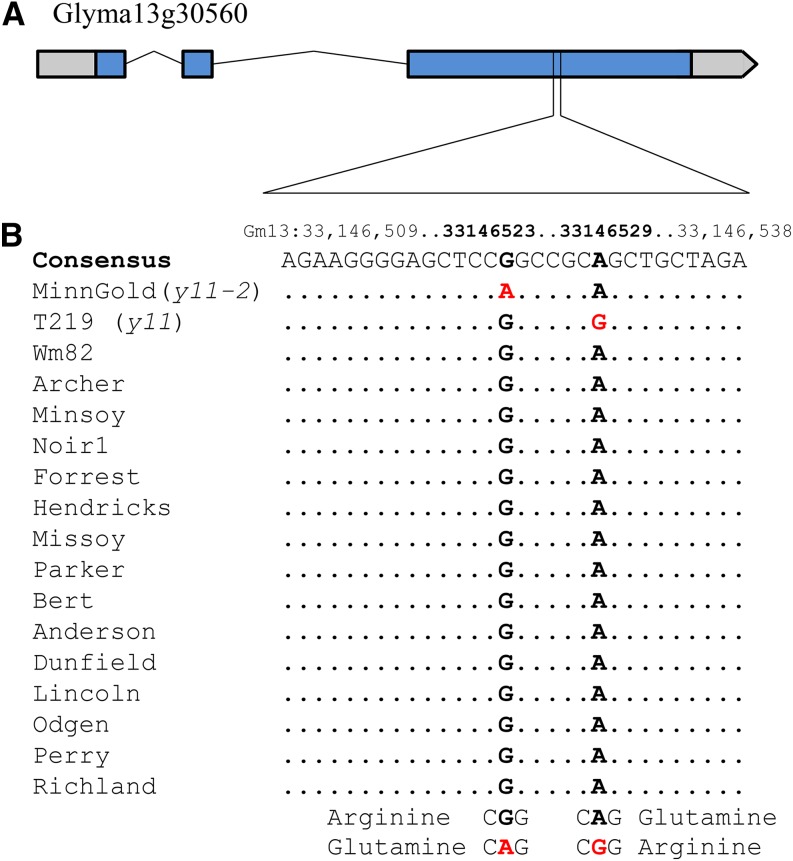
The *y11-2* and *y11* mutations in the candidate gene Glyma13g30560 appear to be novel *de novo* mutations. (A) Gene diagram for the candidate gene Glyma13g30560: the 3 exons are indicated in blue, and 5′ untranslated region (UTR) and 3′ UTR are in gray. (B) Sequence results of MinnGold (*y11-2*) and T219 (*y11*) compared with a panel of diverse soybean lines. Of the 29 diverse soybean lines sequenced (15 are shown), only *y11-2* was found to have adenine rather than guanine at position 33146523 and only *y11* was found to have the guanine rather than the adenine at position 33146529. The *y11-2* SNP causes a nonsynonymous change from arginine to glutamine, and the *y11* SNP causes a nonsynonymous change from glutamine to arginine.

The candidate gene Glyma13g30560 was sequenced in MinnGold and compared with the reference genome sequence (Schmutz *et al.* 2010). A single, nonsynonymous substitution identified in the third exon results in an amino acid substitution of Arginine to Glutamine (R273Q) (Figure 3). A comparative sequence analysis of Glyma13g30560 across twenty-nine diverse soybean lines (McHale *et al.* 2012) found that the single base substitution was unique to MinnGold. All 29 of the diverse lines exhibited the wild-type sequence (Figure 3). The spontaneous occurrence of the mutant phenotype and the unique nature of this specific mutation suggest that this allele appeared *de novo* within the breeding population.

### Transgenic validation of the ChlI1a Mg-chelatase subunit

**Figure 4:**
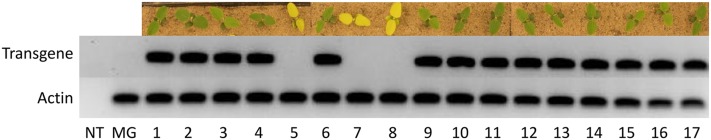
A Glyma13g30560 wild-type transgene complements the chlorophyll deficiency phenotype. The top panel shows the phenotype of 17 T_1_ plants segregating for the presence of the Glyma13g30560 wild-type transgene. NT indicates the no template negative control. MG indicates untransformed MinnGold. The perfect correlation between the presence of the transgene and wild-type phenotype in the segregating T_1_ progeny indicates that the transgene is restoring wild-type function.

The full Glyma13g30560 gene model was cloned from a PCR product amplified from the wild-type cv. ‘Williams 82’. The construct, which contained the genomic sequence spanning from 777 bp upstream of the 5′ UTR to 965 bp downstream of the 3′ UTR, was transformed into MinnGold to test for phenotypic complementation. A successfully transformed T_0_ plant displayed the wild-type green foliage phenotype. Southern blot analysis indicated that this plant harbored a single transgenic locus (data not shown). The subsequent T_1_ progeny segregated in a ratio of 39 green plants to 11 yellow plants, following a 3:1 segregation ratio consistent with a single transgenic locus (χ^2^ test, *P* = 0.62). PCR analysis of the segregating progeny found that the transgenic locus perfectly cosegregated with the green phenotype (Figure 4). This transgenic complementation further indicates that the mutation in the coding region of Glyma13g30560 is responsible for the MinnGold phenotype. Chlorophyll levels were measured in the second true leaf at the V5 growth stage of several MinnGold, ‘Williams 82’, and segregating T_1_ progeny to quantitatively assess the phenotypic effect of the transgene. The chlorophyll levels of wild-type ‘Williams 82’ and transgenic MinnGold plants were not found to be different (*P* = 0.26), whereas the transgenic MinnGold individuals had significantly greater chlorophyll than the untransformed MinnGold line (*P* < 0.0001), thus indicating that the transgene successfully recovered the wild-type phenotype (Figure 1B).

### Sequence analysis suggests the mutation in MinnGold is allelic to y11

The combination of mapping results by Mahama and Palmer (2003) and Zabala and Vodkin (2007) revealed the coincidental mapping of *y11*, another chlorophyll deficient mutant (Figure S1A) to the same chromosome arm as the mutation in MinnGold, and led us to consider that *y11* may also be caused by a mutation in Glyma13g30560. Homozygous mutant (*y11/y11*) plants of the mutant T219H can grow through the seedling stage, but do not survive to set seed. Therefore, a family derived from a single heterozygous T219H plant (*Y11/y11*) was grown to collect DNA from homozygous mutant individuals. Sequencing of Glyma13g30560 from two homozygous chlorophyll deficient yellow mutants (*y11/y11*) revealed a single nonsynonymous SNP change of adenine to guanine in the third exon of Glyma13g30560 [resulting in a change of glutamine to arginine (Q275R)], six base pairs downstream from the SNP change in MinnGold (Figure 3). Glyma13g30560 sequence from two homozygous dark green individuals (*Y11/Y11*) derived from the same segregating T219H family indicated that both of these individuals had the wild-type sequence. A CAPS assay of nineteen individuals segregating for the presence of the candidate *y11* SNP found that the SNP perfectly cosegregated with the foliage phenotype (Figure S2). These results indicate that the mutation in MinnGold would be allelic to *y11*. Therefore, the causative mutation in MinnGold has been assigned the trait designation *y11-2*.

### Identification of identical yellow foliage mutations at paralogous magnesium chelatase genes

**Figure 5:**
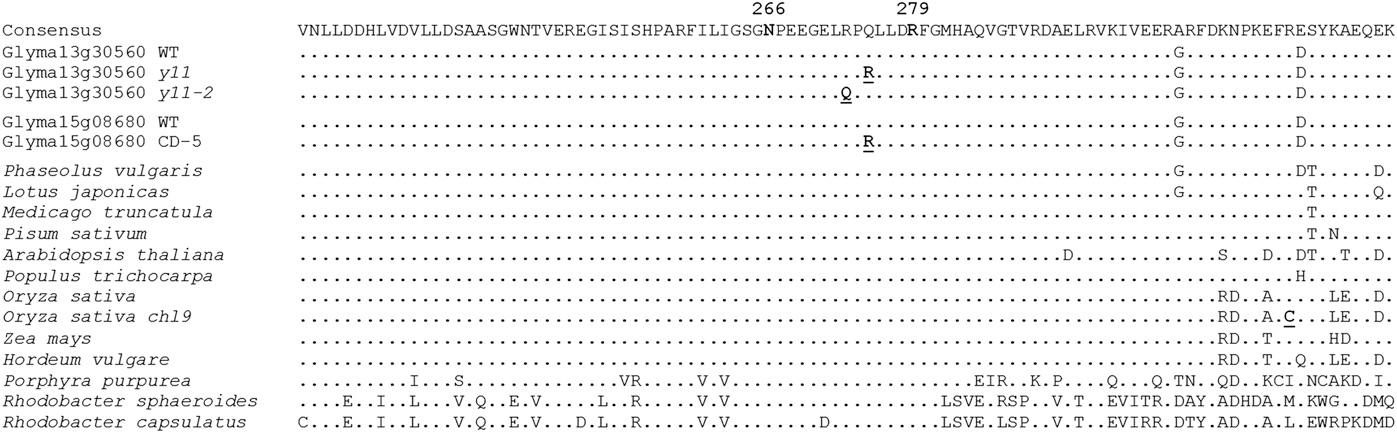
Polypeptide sequence alignment of the Mg-chelatase ChlI subunit for the interval surrounding the *y11*, *y11-2*, and CD-5 mutations. The 266N and 279R positions (bold and labeled in the Consensus sequence) indicate the amino acids predicted to be involved in an ATP binding domain and an arginine binding domain, respectively (Marchler-Bauer *et al.* 2013). The bold and underlined 273Q and 275R positions denote the *y11-2* and *y11* nonsynonymous amino acid changes in Glyma13g30560, respectively. The lower 275R position (in the ‘Glyma15g08680 CD-5′ sequence) denotes the CD-5 nonsynonymous amino acid change in Glyma15g08680. The bold and underlined 313C denotes the nonsynonymous amino acid change in *Oryza sativa chl9*. The letters indicate residues that are not completely conserved across species. The wild-type soybean sequence and the sequences of the other species, listed in order, are available in the National Center for Biotechnology Information database under accession numbers: XP_003543008, XP_003546019, XP_007148073, AFK38677, XP_003593716, AET86637, NP_193583, XP_002316838, NP_001050493, ACN32024, AAA99720, U38804, AF017642, and Z11165.

Palmer *et al.* (1989) found that the *y11* and CD-5 chlorophyll deficient mutants display nearly identical chlorophyll deficient phenotypes (Figure S1). Allelism test results and similarity in phenotype led the authors (Palmer *et al.* 1989) to initially consider that the two mutants were allelic and thus were surprised to find that CD-5 cosegregated with the blunt pubescence tip locus (*pb*) on chromosome 15. After identifying the likely *y11* causative mutation in a Mg-chelatase subunit, we identified a Mg-chelatase paralog on chromosome 15, Glyma15g08680, as a candidate gene for the CD-5. Sequencing of Glyma15g08680 from two homozygous CD-5 individuals and from two wild-type individuals all derived from a segregating family identified a single non-synonymous SNP in the third exon. A CAPS assay of seventeen individuals segregating for the presence of CD-5 showed prefect cosegregation of the candidate SNP with the mutant phenotype (Figure S3). These results suggest that the identified SNP is causative of the CD-5 chlorophyll-deficient phenotype. A comparative sequence analysis across 29 diverse soybean lines (McHale *et al.* 2012) found that the single base substitution in Glyma15g08680 was unique to CD-5. All 29 of the diverse lines exhibited the wild-type sequence (Figure S4A). Remarkably, the Glyma15g08680 amino acid change in CD-5 (Q275R) was identical in position and sequence to the Glyma13g30560 change in *y11* (Q275R) (Figure 5 and Figure S4B).

### Sequence comparison of the Mg-chelatase subunit across photosynthetic species

The clustering of the altered amino acids in the *y11*, *y11-2*, and CD-5 mutants suggests that the affected residues are located in a domain critical for protein function. A comparison of the amino acid sequences of Mg-chelatase ChlI subunit revealed remarkable conservation across angiosperm species (Figure 5). Furthermore, the three nonsynonymous substitutions found in *y11*, *y11-2*, and CD-5 occur at residues that are completely conserved across diverse photosynthetic species, suggesting that these positions are critical for proper ChlI function.

## Discussion

Mg-chelatase is a protein complex comprising three subunits (ChlI, ChlD, and ChlH) that catalyze the insertion of Mg^2+^ into the protoporphyrin IX as the first committed step in the chlorophyll biosynthesis pathway. These three subunits are highly conserved and have been found in all photosynthetic organisms. Both *in vitro* and *in vivo* evidence from bacteria to higher plants have shown that all three components are essential for proper Mg-chelatase function (Gibson *et al.* 1995; Hansson *et al.* 1999, 2002). In soybean, earlier work on the Mg-chelatase ChlI subunit has been limited to molecular characterization, including sequence description, cellular localization, and expression of the putative ChlI (Nakayama *et al.* 1995).

The data presented here show that the T219H and MinnGold chlorophyll-deficient phenotypes perfectly cosegregate with nonsynonymous SNPs located in the third exon of the gene model Glyma13g30560, a Mg-chelatase subunit ChlI1a homolog. We also show that the CD-5 chlorophyll-deficient phenotype perfectly cosegregates with a nonsynonymous SNP located in the third exon of the gene model Glyma15g08680, a Mg-chelatase subunit ChlI1b homolog. The altered residues in *y11*, *y11-2*, and CD-5 are in positions that are conserved across a diverse panel of photosynthetic species, as well as a diverse panel of soybean accessions. Furthermore, genetic transformation of the wild-type Glyma13g30560 allele into the MinnGold background recovered the green foliage (wild-type) phenotype. Collectively, these data provide sufficient evidence to conclude that the chlorophyll deficiency phenotypes of T219H, MinnGold, and CD-5 are caused by mutations in paralogous genes encoding Mg-chelatase subunit ChlI1a (*y11* and *y11-2*) and ChlI1b (CD-5).

In addition to the similar phenotypes exhibited by the *y11* and CD-5 mutants, it was found that these alleles have identical amino acid substitutions (Q275) that occur in the different paralogs. The mutations occurred in two different lines (T219H and CD-5) rather than in a single line, suggesting that gene conversion was not causative of the mutation. To our knowledge, this is the first time that identical mutations have been identified in different paralogs in two phenotypically similar mutant plants.

A high level of residue conservation is seen across species for the Mg-Chelatase subunit as a whole, and the complete amino acid conservation observed specifically at the region in which the *y11*, *y11-2*, and CD-5 mutations occur suggests a high degree of specificity is required for proper functioning (Figure 5). Even the four putative Mg-Chelatase ChlI subunit paralogs of soybean share identical amino acid residues at the mutated positions (data not shown). The amino acid substitutions of *y11* and *y11-2* are separated by only two residues (Q275R and R273Q, respectively), suggesting that these mutations may affect a similar domain in the ChlI1a subunit (Figure 5). The occurrence of the CD-5 mutation, also in this interval but in a separate paralog, further indicates that mutations in this interval disrupt a domain critical for ChlI function (Figure 5 and Figure S5). The *y11*/CD-5 and *y11-2* mutations are seven and nine residues downstream of a predicted ATP binding domain, respectively, and four and six residues upstream of a predicted Arginine finger domain, respectively (Marchler-Bauer *et al.* 2013). Additionally it is predicted that the *y11-2* mutation occurs at a residue immediately preceding an alpha helix domain, while the *y11*/CD-5 mutation occurs within an alpha helix domain (Kelley and Sternberg 2009). However, it is not yet known if these mutations disrupt the ATP binding domain and/or the Arginine finger domain.

**Figure 6:**
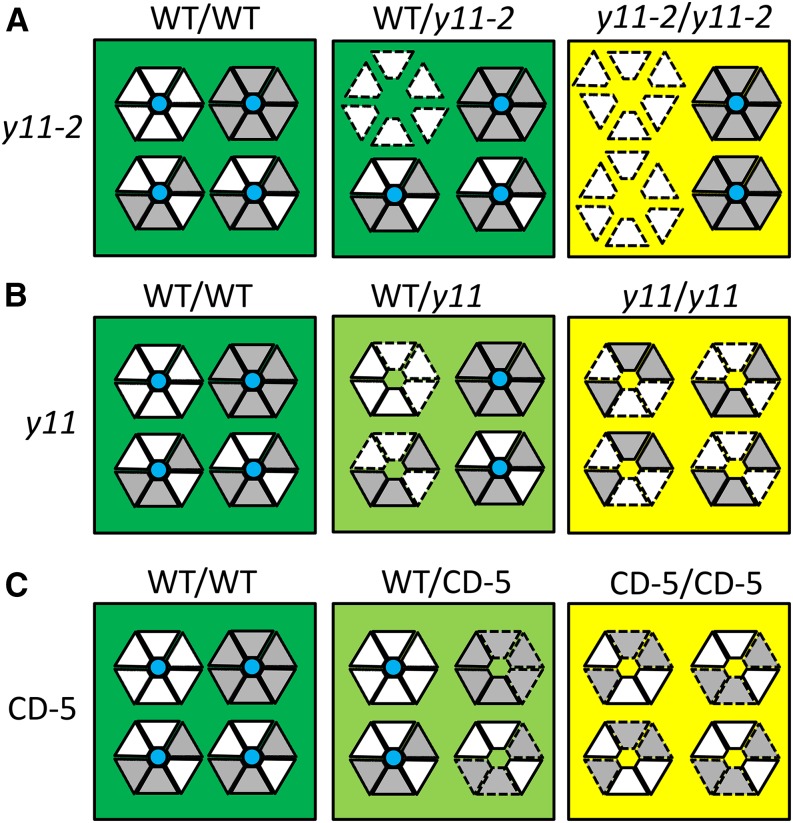
Speculated combinations of *y11-2* (A), *y11* (B), and CD-5 (C) mutant and wild-type ChlI1a and ChlI1b subunits arranged into hexameric rings. The four hexameric rings in each box depict four of the many possible combinations of the ChlI1a (white triangles) and the ChlI1b (gray triangles) subunits assembling together to form the hexameric ring. The resulting chlorophyll phenotypes from the various combinations of wild-type and mutant proteins are indicated by the green, light-green, or yellow background colors. Solid lines around the subunits indicate wild-type proteins, whereas dashed lines around the subunits indicate mutant proteins. A blue circle in the center of the hexamer indicates the hexamer is capable of wild-type activity.

The phenotypes of *y11* and CD-5 are different than *y11*-2, despite being caused by nonsynonymous mutations that are only two amino acid residues apart (Figure 5 and Figure 6), suggesting that there is a high level of specificity required for proper interaction and function of the Mg-chelatase subunits. Interestingly, the *y11* and CD-5 alleles are semidominant whereas the *y11*-2 allele is completely recessive. The semidominance of the *y11* and CD-5 alleles follows what has been observed in other species, where missense ChlI mutations have resulted in semidominant phenotypes (Nguyen 1995; Jensen *et al.* 1996, Hansson *et al.* 1999, 2002; Soldatova *et al.* 2005; Sawers *et al.* 2006; Huang and Li 2009). In contrast, previous studies have found that completely recessive ChlI alleles tend to be caused by presumably more detrimental molecular alterations that truncate the gene or decrease expression (Fischerova 1975; Koncz *et al.* 1990; Rissler *et al.* 2002; Sawers *et al.* 2006; Huang and Li 2009; Kobayashi *et al.* 2008). This appears to be contradictory, as the stronger phenotypic effect (semidominant as opposed to recessive) is associated with a presumably less influential alteration (*e.g.*, an in-frame base substitution as opposed to a gene knockout).

The semidomiant nature of the missense alleles is predicted to be a result of inhibitory interactions between the mutant and wild-type ChlI subunits. Fodje *et al.* (2001) found that the *R. capsulatus* Mg-chelatase subunit orthologous to ChlI1 assembles into a hexameric ring structure complex, and Hansson *et al.* (2002) demonstrated that for the complex to have proper activity, each segment in the hexamer needs to be capable of ATP hydrolysis. Hansson *et al.* (2002) and Sawers *et al.* (2006) demonstrated that the hexameric ring ATPase activity is detrimentally affected when the full-length mutant proteins are assembled into the hexameric ring with the wild-type proteins. The semidominant alleles are thus a result of the mutant ChlI proteins, which do not have full ATPase activity, impeding the function of the wild-type ChlI subunits in the hexameric ring. Therefore, the semidominance of the *y11* and CD-5 alleles identified in this study indeed fit this model.

The *y11-2* allele, however, does not fit this model. This allele is based on a missense mutation at nearly the same location as *y11* and CD-5 but exhibits a completely recessive phenotype. One similar finding has been reported in rice, where a *chl9* missense mutation caused a recessive phenotype, however the position of this mutation was located 40 residues downstream of the *y11-2* mutation in a region with reduced amino acid conservation (Zhang *et al.* 2006) (Figure 5). Still, it is unclear why the *y11-2* allele is recessive whereas the *y11* and CD-5 alleles are semidominant as all three missense mutations occur in the same domain in the ChlI subunit, change the amino acid charge, and affect conserved residues. It is possible that the *y11-2* missense mutation does not interfere with the wild-type ChlI proteins. Instead, the *y11-2* mutation may impede the integration of the mutant ChlI1a protein into the hexameric ring and thus the mutant ChlI protein cannot inhibit the function of wild-type ChlI1a and ChlI1b proteins (see wild-type/*y11-2* and *y11-2/y11-2* in Figure 6A). Thus, it is suggested that the *y11-2* mutant proteins are effectively not involved in chlorophyll biosynthesis. A recessive chlorophyll deficient Arabidopsis mutant, *chli1/chli1* (SAIL_230_D11), which does not express ChlI1 (Huang and Li 2009), displays a phenotype remarkably similar to the *y11-2* phenotype. Although the two mutations are caused by different mechanisms, the common result between the two mutants suggests that neither produces a ChlI1 subunit that is involved in chlorophyll biosynthesis. As a result, both mutants produce a significantly lower level of chlorophyll than wild-type (Huang and Li 2009). For both the *chli1/chli1* and the *y11-2* mutant, paralagous activity (by CHLI2 in Arabidopsis and ChlI1b (Glyma15g08680) in soybean) can partially rescue the phenotypes, assuming neither mutant produces a ChlI that interferes with CHLI2 or ChlI1b function, respectively (Huang and Li 2009).

Additional duplication of the ChlI genes in soybean adds another layer of complexity to understanding the genetics and interactions of this gene family. The paleopolyploid genome has retained four copies of ChlI, including two ChlI2 genes (Glyma07g32550 and Glyma13g24050; Figure S6). The presence of higher-order duplicated gene pairs could provide additional opportunities for sequence evolution through nonfunctionalization, subfunctionalization, or neofunctionalization of the gene paralogs (Force *et al.* 1999; Lynch *et al.* 2001). However, sequence evolution can be constrained in proteins that are part of protein complexes (Fraser *et al.* 2002; Szklarczyk *et al.* 2008). One might hypothesize that the ChlI2 gene copies may influence the ChlI1 mutant phenotypes, either by masking protein malfunctions or contributing additional inhibitory interactions. However, previous work (Severin *et al.* 2010) found that soybean ChlI1a (Glyma13g30560) and ChlI1b (Glyma15g08680) are expressed at much greater levels than the ChlI2a and ChlI2b genes, particularly in leaf tissue (Table S6). Therefore, it is possible that the ChlI2 gene copies have minimal influence on the observed mutant phenotype.

In summary, this study has identified genetic mutations of soybean ChlI1 alleles that confer chlorophyll-deficient foliage phenotypes. Identical missense substitutions at paralogous gene copies were found to confer nearly identical semi-dominant mutant phenotypes, whereas a similar missense mutation *y11-2* conferred a completely recessive phenotype. We speculate that the soybean ChlIa paralogous proteins interact with one another, and the contrasting phenotypes observed from mutations a few base pairs apart may demonstrate the high level of specificity required for these interactions. The delicate nature of this interaction, along with conserved gene function, may contribute to the high sequence conservation of this duplicate gene family. Extended more broadly, the sensitivity of paralogous gene interactions may be crucial in determining whether the duplicate copies are amenable to divergence, or recalcitrant to genetic and transcriptional alterations.

## Supplementary Material

Supporting Information
