# Supplementary material for: Identical Substitutions in Magnesium Chelatase Paralogs Result in Chlorophyll-Deficient Soybean Mutants
Source: G3 (Bethesda). 2014 Dec 1;5(1):123–31. doi: 10.1534/g3.114.015255 (PMC4291463; doi:10.1534/g3.114.015255)
Supplement: Supporting Information [file supp_5_1_123__index.html]

Identical Substitutions in Magnesium Chelatase Paralogs Result in Chlorophyll Deficient Soybean Mutants — Identical Substitutions in Magnesium Chelatase Paralogs Result in Chlorophyll-Deficient Soybean Mutants — Supporting Information 

# Identical Substitutions in Magnesium Chelatase Paralogs Result in Chlorophyll-Deficient Soybean Mutants

## Supporting Information for Campbell *et al.*, 2015

**Files in this Data Supplement:**

- Supporting Information - Tables S1-S6 and Figures S1-S6 (PDF, 306 KB)
- Table S1 - Primer sequences for Sequenom MassARRAY assays used for the first round of fine-mapping for the MinnGold mutation. (PDF, 113 KB)
- Table S2 - Primer sequences for Sequenom MassARRAY assays used for the second round of fine-mapping for the MinnGold mutation. (PDF, 117 KB)
- Table S3 - PCR Primers used to amplify Glyma13g30560 for *y11* and *y11-2*. (PDF, 139 KB)
- Table S4 - PCR Primers used to amplify Glyma15g08680 for CD-5. (PDF, 111 KB)
- Table S5 - Twenty-two genes models present in the fine-mapped interval Gm13: 33,141,206..33,306,556. (PDF, 112 KB)
- Table S6 - Soybean CHLI Genes and Expression Data. (PDF, 111 KB)
- Figure S1 - Phenotypic classes for chlorophyll deficiency mutants. (PDF, 137 KB)
- Figure S2 - *y11* CAPS assay. (PDF, 154 KB)
- Figure S3 - A Cleaved Amplified Polymorphic Sequences (CAPS) assay of seventeen individuals segregating for the presence of the candidate CD-5 SNP. (PDF, 149 KB)
- Figure S4 - The CD-5 mutation in the candidate gene Glyma15g08680 appears to be a novel *de novo* mutation. (PDF, 130 KB)
- Figure S5 - Amino acid sequence comparison of ChlI1a (Glyma13g30560) to ChlI1b (Glyma15g08680) showing the high degree of similarity between the two Mg-chelatase subunits. (PDF, 114 KB)
- Figure S6 - The inferred evolutionary history for the four Glycine Max ChlI subunits calculated using the UPGMA method. (PDF, 116 KB)
