# Supplementary material for: Identical Substitutions in Magnesium Chelatase Paralogs Result in Chlorophyll-Deficient Soybean Mutants
Source: G3 (Bethesda). 2014 Dec 1;5(1):123–31. doi: 10.1534/g3.114.015255 (PMC4291463; doi:10.1534/g3.114.015255)
Supplement: Supporting Information [file supp_g3.114.015255_015255SI.pdf]

G3 · Genes | Genomes | Genetics

Identical substitutions in magnesium chelatase paralogs result in chlorophyll deficient soybean mutants

Benjamin W. Campbell[*], Dhananjay Mani[*], Shaun J. Curtin[*], Rebecca A. Slattery[§], Jean-Michel Michno[*], Donald R. Ort[§,†], Philip J. Schaus[*], Reid G. Palmer[‡], James H. Orf[*], Robert M. Stupar[*]

[*]Department of Agronomy and Plant Genetics, University of Minnesota, St. Paul, MN 55108, U.S.A., [§]Department of Plant Biology, University of Illinois, Urbana, IL 61801, U.S.A., [†]U.S. Department of Agriculture/Agricultural Research Service, Global Change and Photosynthesis Research Unit, Urbana, IL 61801, U.S.A., [‡]Department of Agronomy, Iowa State University, Ames, IA, 50011, U.S.A.

Corresponding Author:
Robert M. Stupar
Department of Agronomy & Plant Genetics
University of Minnesota
1991 Upper Buford Circle
411 Borlaug Hall
St. Paul, MN 55108
Phone: +1-612-625-5769
Email: stup0004@umn.edu

DOI: 10.1534/g3.114.015255

Table S1 Primer sequences for Sequenom MassARRAY assays used for the first round of fine-mapping for the MinnGold mutation.

SNP_ID	2nd-PCRP	1st-PCRP	UEP_SEQ
Gm13_19279455_A_G	ACGTTGGATGTGAACATTGCCTGCTAAGCG	ACGTTGGATGTTTTTGGGCTTGGTGTGAAG	ccccCCAATTAGATCTGCAGAAC
Gm13_23724744_A_G	ACGTTGGATGACGTTTCACCCATGAACACC	ACGTTGGATGCTAGTACTCGGATATTCAGC	ccTCCACTCACCAGTATCA
Gm13_24148253_C_T	ACGTTGGATGCATGACATGTTGAGCCAACC	ACGTTGGATGTGATTGGTTGCGCATGTGAC	TGAGCCAACCTAGTCAT
Gm13_25508582_A_G	ACGTTGGATGCGTCTAGACTCTTTCTGTTC	ACGTTGGATGTATGTACTGACCTCTCATGC	TGCTTGTATCTCATAGAGATAAATAAAC
Gm13_25737735_G_A	ACGTTGGATGGTATGGTTTGTATATTGTGG	ACGTTGGATGTATACTCCAGCGAAGTTCCC	acatgTTGTATATTGTGGACCAACC
Gm13_26671230_A_G	ACGTTGGATGGGCATTTTTGTTGCTGCAGA	ACGTTGGATGGCATGTGAGCACTTGCATTC	TTTTGTTGCTGCAGAAAATAAAT
Gm13_27212330_G_A	ACGTTGGATGAGCAAAGCAGCACTTGCATC	ACGTTGGATGCCATTGGTCGATTGTACTGG	ccccCGTGAACGTCCTCCGGGCAAA
Gm13_27413980_G_A	ACGTTGGATGTCTTTTTGATTGTTTTGTTG	ACGTTGGATGCTTGAGAATATTTACAATCC	TTTTAATTGTAAAGTTCAAAAACCTA
Gm13_27781688_C_A	ACGTTGGATGCTATCTGCAATGATCTTTTC	ACGTTGGATGTCAGCAATCTACTGCAGTTG	TGCAATGATCTTTTCAAGCCTA
Gm13_28465103_C_T	ACGTTGGATGTTTACGTTGGTACCTCCGAA	ACGTTGGATGACATTCTACATCGATCACGC	gGGTACCTCCGAATAACTG
Gm13_28812235_A_G	ACGTTGGATGCCGACTATCGTAGTGAGATG	ACGTTGGATGTCGGCCCCTCATATGATAAG	GATGTTGATTCGTCTATGTCTAAA
Gm13_29565413_G_A	ACGTTGGATGGCAGAACAACTCATGTATCG	ACGTTGGATGCTTTTATGCCAACAAGTGGG	ggatgACAATGCCAATCACTAAGAAAAA
Gm13_29946365_G_A	ACGTTGGATGTGTGGGTATGACCAATCTCC	ACGTTGGATGAAGAGCTGATCCTAACTCCC	gcacTCTCCCATTGTGAAACAACAAGG
Gm13_30268845_G_A	ACGTTGGATGCAGCCTTTGCGCACATCATC	ACGTTGGATGGATCAATGGTATGTTTGTCC	CACATCATCACCCGC
Gm13_30581811_C_T	ACGTTGGATGTACACCGCTAGCTACTTCAC	ACGTTGGATGGTATGGACTTGAGGAAATGG	CTAGCTACTTCACAGGTAA
Gm13_31595360_C_T	ACGTTGGATGTTCACGATGGTGCTTGCAAC	ACGTTGGATGACGACGGTTTTGGAGTTAGC	CAGTCCGAACGGTAG
Gm13_32120834_T_C	ACGTTGGATGCTCCCAAAGTTTCCAATCGG	ACGTTGGATGTCCATGGACAACAACATCCG	CAAGCCATCGGTTTCC
Gm13_33031727_T_C	ACGTTGGATGCAATGGAGCTTATTAAGAAGG	ACGTTGGATGTTCCCTTCACATCCTTGGAG	GCTTATTAAGAAGGTATGCCAG
Gm13_33302559_G_A	ACGTTGGATGAACGCGGTGGTAATCATTGG	ACGTTGGATGAGGGTTCACTCCCAGACCTT	gggagTGTGCCACGTGGTAAAAAG
Gm13_33471044_G_A	ACGTTGGATGCAACAAGTGATCTCGACTCC	ACGTTGGATGTGACACAAGAAAGAAAATC	ggtgACGAGTCAAAGCTAAAGAATAA
Gm13_34624477_T_C	ACGTTGGATGTTGAGCAGCGGCTAAAAGAG	ACGTTGGATGAGACAGCTTTTATCCCATCC	gtgatCAGCGGCTAAAAGAGAGGCCA
Gm13_34778592_C_T	ACGTTGGATGCGTACGTAATTTTAGGAGGC	ACGTTGGATGATTGCTGCTTTCATTATCCC	ggatGAGGCGCGTTTTTATCGACTG
Gm13_35053712_C_A	ACGTTGGATGTCAGGAATTGGTAGGTCAGG	ACGTTGGATGCTCTGCCATTACTGCCAAAG	gGGTCAGGCACAGTAAC
Gm13_35188028_A_G	ACGTTGGATGAACACAACCGCTTTTCTCAC	ACGTTGGATGTGAAGGGCTTATGGAGAGTC	ctcgTTTCTCACAAGCATCCTCTG
Gm13_35492939_T_C	ACGTTGGATGCAAAACCTAACCGCACACTG	ACGTTGGATGTTTGTTGCCGCTCTCATCAC	tcccGCTACGCACAACAAAAATAACCCA
Gm13_36007185_T_C	ACGTTGGATGCCTTGGCCAAACTTATCCTC	ACGTTGGATGGAAAGTTGTCGATGGTGGTG	ccctAGTCAACTCAACCTCAC
Gm13_36188290_G_A	ACGTTGGATGGAGACACGTAGATGGAACAC	ACGTTGGATGAGTGTGTGAGGGAAGATTCG	ccctATGGAACACATTAACACAGCGAG
Gm13_36252057_G_A	ACGTTGGATGATACAATTGAAAGCAGGGAC	ACGTTGGATGGCAGATGGTTGGTATGAAAAG	cGGGAGAAAGGGTATGG
Gm13_36445455_T_C	ACGTTGGATGACTCTTCCCTCATGCTGATG	ACGTTGGATGACTTGTCTCCTTACACCCAC	ACCTTTAAGCTCATAGAGCAT
Gm13_38030960_T_C	ACGTTGGATGCATCAGCATACCTTGAACAG	ACGTTGGATGCCTGTTTGCACATGTTCCAT	ACAGAAAGACAAAGGTTATG

Table S2 Primer sequences for Sequenom MassARRAY assays used for the second round of fine-mapping for the MinnGold mutation.

SNP_ID	2nd-PCRP	1st-PCRP	UEP_SEQ
Gm13_32186965	ACGTTGGATGACTGACTTTTTCCCTTTCG	ACGTTGGATGCAGTGACGATAATTTTAAAGC	actagTTATACTCCCTGTCTCATTATGT
Gm13_32293065	ACGTTGGATGGAGTCTATCCGTTTTATCAC	ACGTTGGATGCGAGTTGCACGTACGAATAC	gtctcTCCGTTTTATCACTTTTACTC
Gm13_32316109	ACGTTGGATGAGGTGTACCTAACAATGCCC	ACGTTGGATGGTAGGTTAGCTAGTGTGTTG	cccaTCACAAGGCTTGGCGTCA
Gm13_32331620	ACGTTGGATGAGAAACATCCGTATGGTTCA	ACGTTGGATGTTTTTGGACTCCGTATGGAC	cccacTCCGTATGGTTCATACAAATT
Gm13_32343536	ACGTTGGATGTTTTAATATTAAGAGATTGG	ACGTTGGATGTATGACAACAGATTTGGTCC	TTTAATATTAAGAGATTGGATTAAGG
Gm13_32365734	ACGTTGGATGGGTGCTTATTTGAGGTTGAG	ACGTTGGATGGATACATAAAGTGAGTCTCT	tccaATATAACTTATTATTTGGCTGGT
Gm13_32586550	ACGTTGGATGGTGGGAGAGTTTTCATATAG	ACGTTGGATGCAAGCTAAGGACTGGTTGAC	cTTAGGAGTTGATTAGCGA
Gm13_32636705	ACGTTGGATGCACTAGGGCAGTGACTTCAT	ACGTTGGATGGGAATGTGCAAGTGTCTCTG	ctTCGCGTGCCTTTTTT
Gm13_32657362	ACGTTGGATGACGGATCACATTGGCTATGG	ACGTTGGATGGTTTCATTCGTGCATGCGAT	CATTGGCTATGGAATTCGATTA
Gm13_32673914	ACGTTGGATGGGTGAATTTGACGGGTGAAC	ACGTTGGATGATTCAGTTTGGGCTTGTCGC	GGTGAACTCAGTGGAA
Gm13_32954871	ACGTTGGATGTCACAGTAGGAGGTGTAGTC	ACGTTGGATGTAAAGTTGCTGCTCCAGCTC	aagatGAGGTGTAGTCGTAGGA
Gm13_32998715	ACGTTGGATGTTTTTCAAGATTGCGCTGGG	ACGTTGGATGAATAGAACCTTCACGGCTTC	ccgcTTGCGCTGGGTTGTGGT
Gm13_33026763	ACGTTGGATGATGCGCTTGAGTGGATGAAC	ACGTTGGATGTCTTCCGGAACTGCTTGTAG	TTGTTGGAAGAGAAAAAGCA
Gm13_33027247	ACGTTGGATGCTCCAGCCAAAGATTCTTCC	ACGTTGGATGCTAGGGTCCAAAATTCTCTG	TAGCCAACTCTATTGCAATAATGG
Gm13_33031727	ACGTTGGATGCAATGGAGCTTATTAAGAAGG	ACGTTGGATGTTCCCTTCACATCCTTGGAG	CTTATTAAGAAGGTATGCCAG
Gm13_33057712	ACGTTGGATGGCTTTATAGATGTTAGCCTTC	ACGTTGGATGGAGGAGAAGGTTGTTTGGTC	ctggTAGCCTTCATCTAAATGTCC
Gm13_33060476	ACGTTGGATGGACGGTTGTTCTTTTGTGGC	ACGTTGGATGCAGTTTCGTAATTACATTG	ccctcTCTTTTGTGGCTAATAAAAACTT
Gm13_33061634	ACGTTGGATGTCACAACATAACGTTGCAGG	ACGTTGGATGTCTAGCGCCACATAGTGAAG	ACGTTGCAGGTACAAAAA
Gm13_33063980	ACGTTGGATGCCTTCTTCGACTCTGCATTC	ACGTTGGATGCCCTCGGAAGTTGTAAAATC	aTCTGCATTCTTCTTCCT
Gm13_33124381	ACGTTGGATGACCATGGAGTCTGCAAGAAC	ACGTTGGATGGGGAGTTTGTCATGAGGATG	AGCAGCCCACACATCC
Gm13_33128103	ACGTTGGATGACATTGATGATTAGGGTGAC	ACGTTGGATGCAATTTACAACTTGTTCGGG	TGTCTAATTATATAAATACATTAGCTG
Gm13_33141206	ACGTTGGATGACAAACTCACAGCAGGAGTC	ACGTTGGATGTGTTGTGTTCTGCGCTTTGG	TATGTCTCTGGGTTTGGTG
Gm13_33146523	ACGTTGGATGCCAACTTGAGCATGCATTCC	ACGTTGGATGTCCTAATTGGCTCAGGCAAC	TCTAGCAGCTGCGGC
Gm13_33151333	ACGTTGGATGGTCACATAAATTGTACTAC	ACGTTGGATGGCTTTCCACTTACTCTGGTC	acAATCTTTAAGCAAACATCAAAT
Gm13_33191293	ACGTTGGATGACATTTGGCTTCCCTAGGTG	ACGTTGGATGCACACTCGCTCAGCGTGAT	aaacCCCTAGGTGGCTTCTTC
Gm13_33206265	ACGTTGGATGGCCTCATTTGGTCAGATATG	ACGTTGGATGGTCCTTATAAAACAACCAGCC	CTTTATTTGCTTATCATCCCTTT
Gm13_33212151	ACGTTGGATGGGAGCTGAAAGGTCTAAGAG	ACGTTGGATGAAAAAAATGCGCTCTCCTCCC	GTCTAAGAGCCTTTAAAACA
Gm13_33251230	ACGTTGGATGAGGTGCGCTTAGCGTAAATC	ACGTTGGATGAAAATTCACGCGATCTCACG	GCTAAACCCAGGAGG
Gm13_33271690	ACGTTGGATGTAAACTCTCTGCCCCTTTCC	ACGTTGGATGAGAAAGCAACTTGAGAGGCG	aagaTATTCTTTCTCTCCACGCTCTA
Gm13_33306556	ACGTTGGATGCTTCTACCAACCTTGATACC	ACGTTGGATGGAAATCCATGAGGGATGTTG	CAACCTTGATACCGCTAAC
Gm13_33309287	ACGTTGGATGTTACTATAAGTTAGTTTGTG	ACGTTGGATGTGCCCTTAAGGTGTTATCCC	TTACTATAAGTTAGTTTGTGTGATTAA
Gm13_33309683	ACGTTGGATGGCTTCAGCTCGTCAAGGAAC	ACGTTGGATGTCAGAATGAAACTCTTGCCC	CGTGGTCTCTGTGTG
Gm13_33429985	ACGTTGGATGCACTCGAATAAAGATCTCGG	ACGTTGGATGTATATGGGCGAGCCAAATTC	AGATCTCGGTTCTAAATCATTG
Gm13_33433989	ACGTTGGATGTTGCTTCTATGACAAACAC	ACGTTGGATGCAGAAAAGAAAAAACAGCCC	ACAAACACAATATTTACTACTTTTTAAT
Gm13_33434371	ACGTTGGATGCCATCATACTCTCACACAGG	ACGTTGGATGAGGTCTTGTTCCTCCTCTTC	ggaagCAGGAAACATTGAGACTC
Gm13_33439666	ACGTTGGATGGGACTAGAAATTAAGCTGTC	ACGTTGGATGGAAGAAATATCACTTTGGAAC	CATTTGTACACACTTGCCT
Gm13_33457770	ACGTTGGATGTTCACGAGTAGGGCAACAAC	ACGTTGGATGAAGAACGTCGTTGCCTATGG	TTCGGGTTACTTCGACTTAG
Gm13_33488403	ACGTTGGATGCCAACATCAATCATCCACCG	ACGTTGGATGATGGGTGGGCTTTAGATGAG	ACCGTCCGATCCTTC
Gm13_33505944	ACGTTGGATGTCACCACACCTTTTCCAACG	ACGTTGGATGGAAAGGTCATTGGAGTTGCG	tttcCAACTTTTCCGGCCATC

Gm13_33563545	ACGTTGGATGCCAAAACTCAAACAGAATG	ACGTTGGATGTGTGTTCATAATTTACCTC	tgGAACTAAAAATCAAGTGTTTAGTA
Gm13_33634870	ACGTTGGATGACAACAAGGTTCTGACCTCG	ACGTTGGATGGTGACCAATGACTTGTAGTG	tcgaCAACATGACCTGCGTTTTGAGC
Gm13_34213468	ACGTTGGATGGTCTTGAAACTTCTCCTCGG	ACGTTGGATGAAGAGGTTCAAGTGCTCCAG	cAGCCAAGCCTACGAC
Gm13_34233496	ACGTTGGATGTGTTAAGTGCAGGAACACTC	ACGTTGGATGTTGAATCCTCGTTTAGCCCG	cggggTAAGTGCAGGAACACTCAACTCT
Gm13_34248212	ACGTTGGATGACTCCTCCACGTGTTGAATC	ACGTTGGATGCGAGAACGGCTTTTACTTGG	gaaaGTGTTGAATCCATGCCCGC
Gm13_34293335	ACGTTGGATGAAAGGCCCATTTCGTGTTAG	ACGTTGGATGAGATCGGGTGGGTTAATTCG	attaTTTTTAATTTTAAAAAGGGCATAG
Gm13_34453742	ACGTTGGATGTCGCTATGTGTCACTTGTGG	ACGTTGGATGAGTTGTCAAGCTGCAATGCG	tcctAGCTCAACAACCTCTG
Gm13_34465720	ACGTTGGATGTTCGCGATGGGTTTCTTCAG	ACGTTGGATGTCTGATCCGGGAATGTTGTC	GCTTCGACAACTCCTC
Gm13_34602307	ACGTTGGATGTTGTTATGGGCCAAAAGTGC	ACGTTGGATGGCCTTATTTCATTACTGTGG	GGGCCAAAAGTGCCAAATTATATC
Gm13_34645498	ACGTTGGATGTTTTAAAAGGGTCGAGTCGG	ACGTTGGATGTGATCCGATTAAATCCGGTC	cccctTCGGACCGGTCTAACCGGGATC
Gm13_34701345	ACGTTGGATGCTTGTGATTTCTGTCTCCTC	ACGTTGGATGTTTGGCCACTTCTTCACCTC	tctaCTCATTTCTCATTTTCATGTG
Gm13_34748233	ACGTTGGATGATTGTCAAATGCACCCGAGC	ACGTTGGATGCTGTGGTGCATCCAATTGTG	AGCTCGATTCATCAAAACTTC
Gm13_34759965	ACGTTGGATGGAATCATCATTCATGTTCG	ACGTTGGATGCATTCTCTCTGTGCAATCCA	ccctAATCATCATTCATGTTCGTTTTAG
Gm13_34778592	ACGTTGGATGCGTACGTAATTTTAGGAGGC	ACGTTGGATGATTGCTGCTTTCATTATCCC	ccccaCGCGTTTTTATCGACTG
Gm13_34801784	ACGTTGGATGCGGGACGGAACTAGAAAAAA	ACGTTGGATGCCTCCAAAGACACTAAAAAG	AAAGAAAAAAATATTGCTTAACTTAT
Gm13_34808743	ACGTTGGATGTCTGTATCCGTGTCGTGTCC	ACGTTGGATGAGAGTTCAGACAATGTGAAG	gggcTCCGGTGTCCATTTCG
Gm13_34810502	ACGTTGGATGAGTTGGATGGCATCCTGAAC	ACGTTGGATGCTACACCATGAGCAGTTGGC	TGATCTATTACTTATTTATGGATAAAGA
Gm13_34819160	ACGTTGGATGTGTCTGCTACTTTTTCCCCC	ACGTTGGATGCTTGTTTCGGGTCACTAGTT	TCCCCCATATTTTGGATA
Gm13_34826532	ACGTTGGATGGCCTCAACGGACATCCAAAG	ACGTTGGATGCCTTTGCGGAATTAGATGGC	ACATCCAAAGAAACCGT
Gm13_34828053	ACGTTGGATGGCTCGAATACATGGACGAGG	ACGTTGGATGGCCTATCATATCCATCTGCG	GGACGAGGTATGCTCAT
Gm13_34831302	ACGTTGGATGAGTACTTAACATCTCCTAC	ACGTTGGATGTTTGTTTGAGGCTGTTTGAC	TTAACATCTCCTACTATCACT
Gm13_34855829	ACGTTGGATGATGTCAGGTGGTTCTAGTGG	ACGTTGGATGACCTCCTCATATTCTGCCTC	tTTCTCAGGGTGGGGA
Gm13_34859558	ACGTTGGATGAGTTATTTGGGGTCTGGTGG	ACGTTGGATGATGCACCTCCAAGGAGAAAC	ggaagGGTGGGACCAAAGTGTAT
Gm13_34861415	ACGTTGGATGTGACCAAGGTGTTTGACCTG	ACGTTGGATGGCCATATCTAGGACTAAAATC	aTCTCAAGTCTCAAGCTAA
Gm13_34862245	ACGTTGGATGGAGAACAACAACAAAGCTAC	ACGTTGGATGGTTACTGTCATAGTGCCCTG	caaaAACAAAGCTACAACAAAGTAA
Gm13_34870402	ACGTTGGATGTTCTGGATCGTGTTCAGGAG	ACGTTGGATGAACCCAGCTTTTAGTTTCCC	CGTGTTCAGGAGGAAATT
Gm13_34871976	ACGTTGGATGCTGAGTTAGGTAGGAATAGG	ACGTTGGATGGACCAACAGTTCAAAACGTC	AGGTAGGAATAGGATCACTTGA
Gm13_34882559	ACGTTGGATGGCCCACTAAATTTGTGGCTA	ACGTTGGATGATGTTGAACGTAATCCTCTC	CAATGTATTGTGAATTGTAATATTATTG
Gm13_34888379	ACGTTGGATGCGCCCTTTCTGTTAAGTGTG	ACGTTGGATGTCTATCCATTCGACACGTGC	ggggtTGTGATTGTGAAAGAATCC
Gm13_34890355	ACGTTGGATGATTATGGTGGTCTCACTCGC	ACGTTGGATGGTCTTACCGTAATTTTGGA	cgagaTGTGCATTGGTAGCATTGCG
Gm13_34895902	ACGTTGGATGTGATCTCAAGGGCATTGGAC	ACGTTGGATGTCCGAGCTTCTCTGACTGTG	gGGAGGTAGCGAACTG

Table S3 PCR Primers used to amplify Glyma13g30560 for *y11* and *y11-2*.

PCR Reaction	Type	Primer Pair First Reaction	Type	Primer Pair Second Reaction (Nested)
1	Forward	TGGCACCCACTAACATTTCC	Forward	TGGCACCCACTAACATTTCC
1	Reverse	CCAGTATCCTTTTTATTTAGGAGACC	Reverse	CCAGTATCCTTTTTATTTAGGAGACC
2	Forward	TGGCACCCACTAACATTTCC	Forward	CGTTTTTGTCTTAAAAGCTTGATT
2	Reverse	CACACAACACACAAAAGAATGG	Reverse	GTTTGCAGCACACCATCC
3	Forward	TGGCACCCACTAACATTTCC	Forward	TCTTCTTCTTCCAAGCCTTCC
3	Reverse	CACACAACACACAAAAGAATGG	Reverse	CACACAACACACAAAAGAATGG
4	Forward	GGCCAGGCCTTTGCATTTTG	Forward	GGCCAGGCCTTTGCATTTTG
4	Reverse	ACTCAGCACACACCTTGGAG	Reverse	ACTCAGCACACACCTTGGAG
5	Forward	CCTGACTGAGGGTGTCAAGG	Forward	CCTGACTGAGGGTGTCAAGG
5	Reverse	GAAGTTAATCTTGGAGTGTATTTTGC	Reverse	GGCACTTACGTTGTCTCTTCC
6	Forward	CCTGACTGAGGGTGTCAAGG	Forward	GCTGAGTTGAATGTGGATGG
6	Reverse	GAAGTTAATCTTGGAGTGTATTTTGC	Reverse	GAAGTTAATCTTGGAGTGTATTTTGC

Table S4 PCR Primers used to amplify Glyma15g08680 for CD-5.

PCR Reaction	Type	Primer Pair First Reaction	Type	Primer Pair Second Reaction (Nested)
1	Forward	CGGAGACTGGTAAATGTGAGC	Forward	CGGAGACTGGTAAATGTGAGC
1	Reverse	CAGCACACCTCCAAAACAAG	Reverse	CAGCACACCTCCAAAACAAG
2	Forward	GGCTAGGCCTTTGTGTTTGA	Forward	GGCTAGGCCTTTGTGTTTGA
2	Reverse	AACGGGAAATGCTGATTGAG	Reverse	AACGGGAAATGCTGATTGAG
3	Forward	GCTGCTGGATAGGTTTGGAA	Forward	GCTGCTGGATAGGTTTGGAA
3	Reverse	AACGGGAAATGCTGATTGAG	Reverse	AACGGGAAATGCTGATTGAG
4	Forward	TCAATTGCGGTTCTTCCTTC	Forward	ACGTCAGCTGCTATGAATGG
4	Reverse	TGGCCTCTGGCTTTCTTTAG	Reverse	GTAGCTCCCAATGGCAAATC
5	Forward	TCAATTGCGGTTCTTCCTTC	Forward	TCAATTGCGGTTCTTCCTTC
5	Reverse	TGGCCTCTGGCTTTCTTTAG	Reverse	TGGCCTCTGGCTTTCTTTAG

Table S5 Twenty-two genes models present in the fine-mapped interval Gm13: 33,141,206..33,306,556

Gene	Location	Gene Annotation
Glyma13g30550	Gm13: 33141000..33143927	Transferase family
Glyma13g30560	Gm13: 33144571..33147208	Magnesium chelatase, subunit ChlI
Glyma13g30575	Gm13: 33147978..33151645	Peroxisomal NUDIX hydrolase
Glyma13g30590	Gm13: 33154582..33158861	Family not named
Glyma13g30600	Gm13: 33163957..33165210	Zinc finger, C3HC4 type (RING finger)
Glyma13g30610	Gm13: 33171016..33182189	ATP-Dependent RNA Helicase
Glyma13g30620	Gm13: 33183067..33188556	Glutamate-gated kainate-type ion channel receptor subunit GluR5 and related subunits
Glyma13g30625	Gm13: 33193010..33194243	There are no functional annotations for this locus
Glyma13g30630	Gm13: 33193058..33193890	There are no functional annotations for this locus
Glyma13g30650	Gm13: 33194700..33195205	There are no functional annotations for this locus
Glyma13g30640	Gm13: 33194530..33201169	Glutamate-gated kainate-type ion channel receptor subunit GluR5 and related subunits
Glyma13g30660	Gm13: 33204225..33209549	Glutamate-gated kainate-type ion channel receptor subunit GluR5 and related subunits
Glyma13g30670	Gm13: 33210247..33214795	D-Tyr-tRNA (Tyr) deacylase
Glyma13g30680	Gm13: 33221124..33226612	GDSL-like Lipase/Acylhydrolase
Glyma13g30690	Gm13: 33231142..33237718	GDSL-like Lipase/Acylhydrolase
Glyma13g30710	Gm13: 33243653..33244787	Regulation of transcription, DNA-dependent
Glyma13g30720	Gm13: 33247690..33248688	Regulation of transcription, DNA-dependent
Glyma13g30730	Gm13: 33259622..33263250	Pterin carbinolamine dehydratase PCBD/dimerization cofactor of HNF1
Glyma13g30740	Gm13: 33263320..33264787	Protein of unknown function, DUF599
Glyma13g30750	Gm13: 33280224..33286703	Auxin response factor
Glyma13g30760	Gm13: 33291706..33297139	Mlo family
Glyma13g30770	Gm13: 33302261..33303185	Glutaredoxin

Table S6 Soybean CHLI Genes and Expression Data. Normalized soybean expression data showing that Glyma13g30560 and Glyma15g08680 share similar expression patterns, however Glyma13g30560 showing approximately twice the expression level in leaf tissues. Source: Severin, AJ, Woody JL, Bolon YT, Joseph B, Diers BW, et al. (2010) RNA-Seq Atlas of Glycine max: a guide to the soybean transcriptome. BMC Plant Biol 10:160.

Gene	Young leaf	Flower	One cm pod	Pod shell 10DAF	Pod shell 14DAF	Seed 10DAF	Seed 14DAF	Seed 21DAF	Seed 25DAF	Seed 28DAF	Seed 35DAF	Seed 42DAF	Root	Nodule
Glyma13g30560	106	16	38	41	33	2	3	6	8	6	8	3	1	1
Glyma15g08680	51	8	16	18	17	2	5	7	9	5	7	3	1	1
Glyma07g32550	2	3	2	1	1	1	1	1	1	0	0	0	2	1
Glyma13g24050	0	2	0	0	1	1	2	0	1	0	1	0	1	1

Figure S1

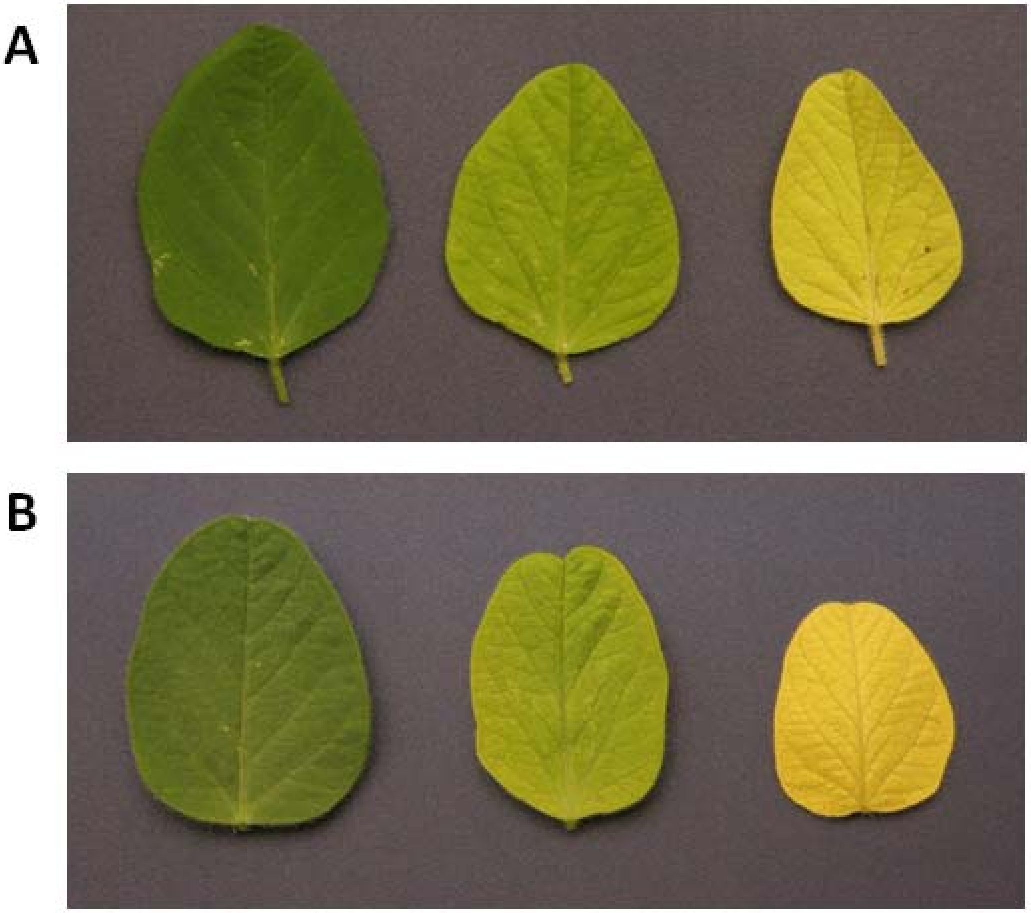

Figure S1 Phenotypic classes for chlorophyll deficiency mutants. (A) Leaves of *Y11/Y11*, *Y11/y11*, and *y11/y11* leaves showing the distinctive phenotypic classes. (B) Leaves of WT/WT, WT/CD-5, and CD-5/CD-5 leaves showing the distinctive phenotypic classes.

Figure S2

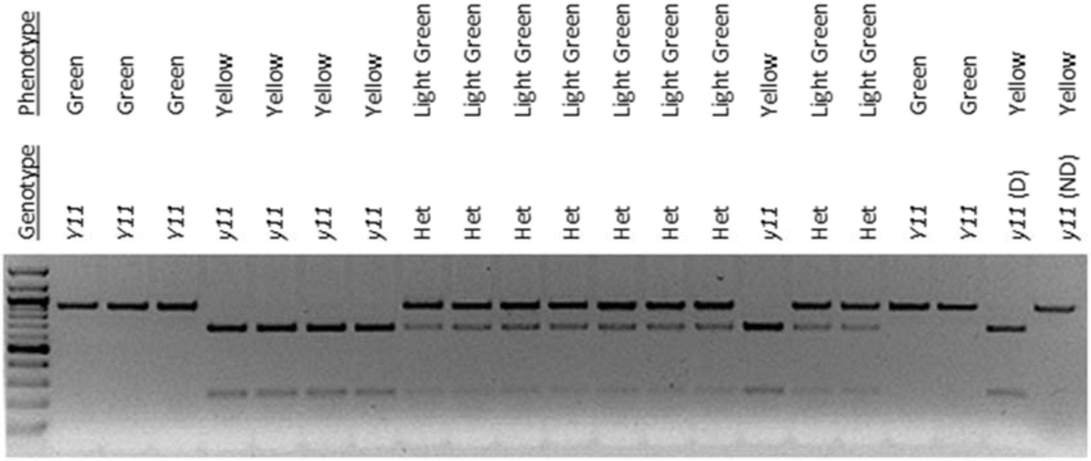

Figure S2 *y11* CAPS assay. A Cleaved Amplified Polymorphic Sequences (CAPS) assay of nineteen individuals segregating for the presence of the candidate *y11* SNP. In the rightmost lanes, D indicates a *y11/y11* digested sample and a ND indicates a *y11/y11* sample that was not digested. The perfect cosegregation of the candidate *y11* SNP with the foliage phenotype provides additional information to suggest that the candidate SNP is responsible for the chlorophyll deficient phenotype.

Figure S3

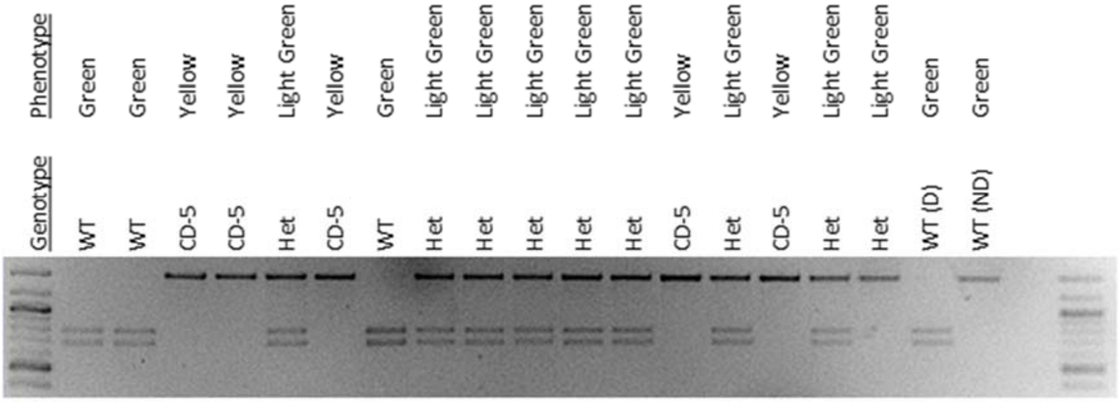

Figure S3 A Cleaved Amplified Polymorphic Sequences (CAPS) assay of seventeen individuals segregating for the presence of the candidate CD-5 SNP. In the rightmost lanes, D indicates a WT/WT digested sample and a ND indicates a WT/WT sample that was not digested. The perfect cosegregation of the candidate CD-5 SNP with the foliage phenotype provides additional information to suggest that the candidate SNP is responsible for the chlorophyll deficient phenotype.

Figure S4

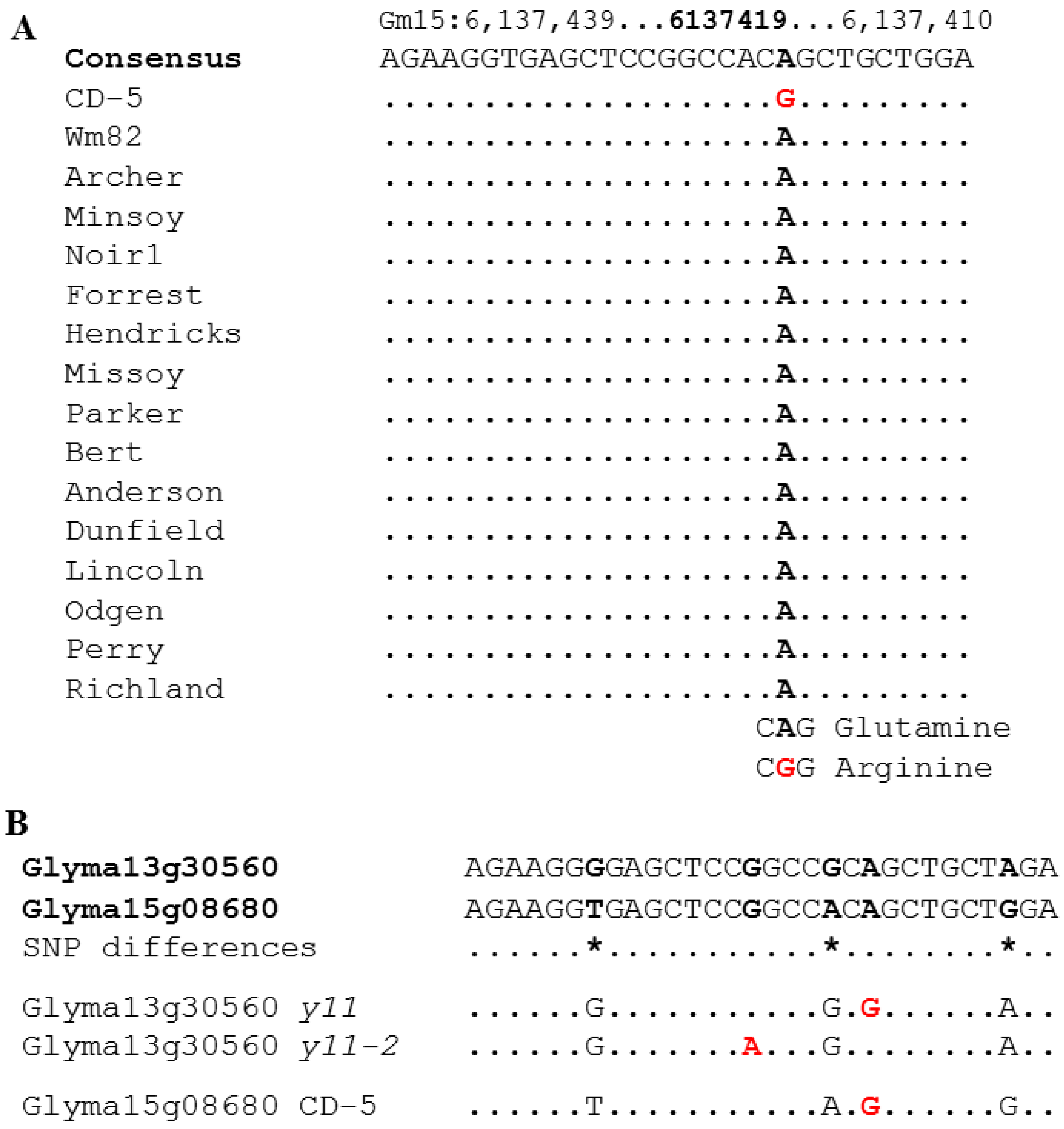

Figure S4 The CD-5 mutation in the candidate gene Glyma15g08680 appears to be a novel *de novo* mutation. (A) Sequence results of CD-5 compared to a panel of diverse soybean lines. Of the 29 diverse soybean lines sequenced (15 are shown), only CD-5 was found to have Guanine rather than Adenine at position 6137419. The CD-5 SNP causes a nonsynonymous change from Glutamine to Arginine. (B) Sequences from Glyma13g30560 and Glyma15g08680 showing that the *y11* and CD-5 SNPs in the separate genes occur at the same relative SNP position, but that sequences from the two genes can be differentiated by nearby SNPs. Sequences for Glyma13g30560 and Glyma15g08680 are from positions Gm13:33,146,509..33,146,538 and Gm15:6,137,439..6,137,410, respectively.

Figure S5

```
Consensus  MAS.LGTSSI AVLPSR..SS .SSKPSIHTL SLTSGQ.YGR KFYGGIGIHG 50
Gma ChlI1a ...A...... ......YF.. S......... ......N... ..........
Gma ChlI1b ...T...... ......CI.. F......... ......S... ..........

Consensus  IKGR.QLSV. NVATEVNSVE QAQSIASKES QRPVYPFSAI VGQDEMKLCL 100
Gma ChlI1a ....A....T .......... .......... .......... ..........
Gma ChlI1b ....S....A .......... .......... .......... ..........

Consensus  LLNVIDPKIG GVMIMGDRGT GKSTTVRSLV DLLPEIKVVA GDPYNSDPQD 150
Gma ChlI1a .......... .......... .......... .......... ..........
Gma ChlI1b .......... .......... .......... .......... ..........

Consensus  PEFMGVEVRE RVLQGEELSV VLTKINMVDL PLGATEDRVC GTIDIEKALT 200
Gma ChlI1a .......... .......... .......... .......... ..........
Gma ChlI1b .......... .......... .......... .......... ..........

Consensus  EGVKAFEPGL LAKANRGILY VDEVNLLDDH LVDVLLDSAA SGWNTVEREG 250
Gma ChlI1a .......... .......... .......... .......... ..........
Gma ChlI1b .......... .......... .......... .......... ..........

Consensus  ISISHPARFI LIGSGNPEEG ELRPQLLDRF GMHAQVGTVR DAELRVKIVE 300
Gma ChlI1a .......... .......... .......... .......... ..........
Gma ChlI1b .......... .......... .......... .......... ..........

Consensus  ERGRFDKNPK EFRDSYKAEQ EKLQQQITSA RSVLSSVQID QDLKVKISKV 350
Gma ChlI1a .......... .......... .......... .......... ..........
Gma ChlI1b .......... .......... .......... .......... ..........

Consensus  CAELNVDGLR GDIVTNRAAK ALAALK.RD. VSAEDIATVI PNCLRHRLRK 400
Gma ChlI1a .......... .......... ......G..N .......... ..........
Gma ChlI1b .......... .......... ......E..K .......... ..........

Consensus  DPLESIDSGL LVTEKFYEVF S 421
Gma ChlI1a .......... .......... .
Gma ChlI1b .......... .......... .
```

Figure S5 Amino acid sequence comparison of ChlI1a (Glyma13g30560) to ChlI1b (Glyma15g08680) showing the high degree of similarity between the two Mg-chelatase subunits. The two boxed residues indicate the positions of the *y11-2* (R273Q) mutation and *y11* and CD-5 (Q275R) mutations.

Figure S6

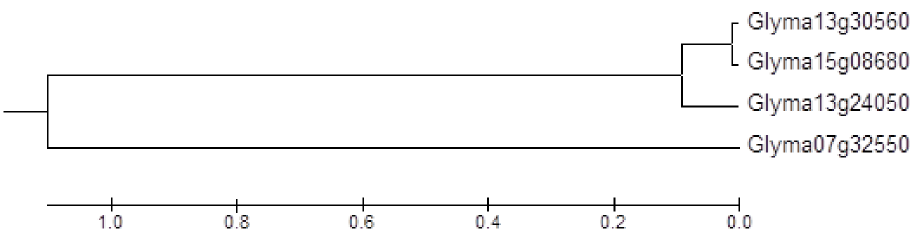

Figure S6 The inferred evolutionary history for the four Glycine Max ChlI subunits calculated using the UPGMA method. The UPGMA neighbor joining tree shows the close sequence similarity of Glyma13g30560 with Glyma15g08680. The optimal tree with the sum of branch length = 2.30757376 is shown.
