# Supplementary material for: Identical Substitutions in Magnesium Chelatase Paralogs Result in Chlorophyll-Deficient Soybean Mutants
Source: G3 (Bethesda). 2014 Dec 1;5(1):123–31. doi: 10.1534/g3.114.015255 (PMC4291463; doi:10.1534/g3.114.015255)
Supplement: Supporting Information [file supp_g3.114.015255_TableS1.pdf]

Table S1 Primer sequences for Sequenom MassARRAY assays used for the first round of fine-mapping for the MinnGold mutation.

SNP_ID	2nd-PCRP	1st-PCRP	UEP_SEQ
Gm13_19279455_A_G	ACGTTGGATGTGAACATTGCCTGCTAAGCG	ACGTTGGATGTTTTTGGGCTTGGTGTGAAG	ccccCCAATTAGATCTGCAGAAC
Gm13_23724744_A_G	ACGTTGGATGACGTTTCACCCATGAACACC	ACGTTGGATGCTAGTACTCGGATATTCAGC	ccTCCACTCACCAGTATCA
Gm13_24148253_C_T	ACGTTGGATGCATGACATGTTGAGCCAACC	ACGTTGGATGTGATTGGTTGCGCATGTGAC	TGAGCCAACCTAGTCAT
Gm13_25508582_A_G	ACGTTGGATGCGTCTAGACTCTTTCTGTTC	ACGTTGGATGTATGTACTGACCTCTCATGC	TGCTTGTATCTCATAGAGATAAATAAAC
Gm13_25737735_G_A	ACGTTGGATGGTATGGTTTGTATATTGTGG	ACGTTGGATGTATACTCCAGCGAAGTTCCC	acatgTTGTATATTGTGGACCAACC
Gm13_26671230_A_G	ACGTTGGATGGGCATTTTTGTTGCTGCAGA	ACGTTGGATGGCATGTGAGCACTTGCATTC	TTTTGTTGCTGCAGAAAATAAAT
Gm13_27212330_G_A	ACGTTGGATGAGCAAAGCAGCACTTGCATC	ACGTTGGATGCCATTGGTCGATTGTACTGG	ccccCGTGAACGTCCTCCGGGCAAA
Gm13_27413980_G_A	ACGTTGGATGTCTTTTTGATTGTTTTGTTG	ACGTTGGATGCTTGAGAATATTTACAATCC	TTTTAATTGTAAAGTTCAAAAACCTA
Gm13_27781688_C_A	ACGTTGGATGCTATCTGCAATGATCTTTTC	ACGTTGGATGTCAGCAATCTACTGCAGTTG	TGCAATGATCTTTTCAAGCCTA
Gm13_28465103_C_T	ACGTTGGATGTTTACGTTGGTACCTCCGAA	ACGTTGGATGACATTCTACATCGATCACGC	gGGTACCTCCGAATAACTG
Gm13_28812235_A_G	ACGTTGGATGCCGACTATCGTAGTGAGATG	ACGTTGGATGTCGGCCCCTCATATGATAAG	GATGTTGATTCGTCTATGTCTAAA
Gm13_29565413_G_A	ACGTTGGATGGCAGAACAACTCATGTATCG	ACGTTGGATGCTTTTATGCCAACAAGTGGG	ggatgACAATGCCAATCACTAAGAAAAA
Gm13_29946365_G_A	ACGTTGGATGTGTGGGTATGACCAATCTCC	ACGTTGGATGAAGAGCTGATCCTAACTCCC	gcacTCTCCCATTGTGAAACAACAAGG
Gm13_30268845_G_A	ACGTTGGATGCAGCCTTTGCGCACATCATC	ACGTTGGATGGATCAATGGTATGTTTGTCC	CACATCATCACCCGC
Gm13_30581811_C_T	ACGTTGGATGTACACCGCTAGCTACTTCAC	ACGTTGGATGGTATGGACTTGAGGAAATGG	CTAGCTACTTCACAGGTAA
Gm13_31595360_C_T	ACGTTGGATGTTCACGATGGTGCTTGCAAC	ACGTTGGATGACGACGGTTTTGGAGTTAGC	CAGTCCGAACGGTAG
Gm13_32120834_T_C	ACGTTGGATGCTCCCAAAGTTTCCAATCGG	ACGTTGGATGTCCATGGACAACAACATCCG	CAAGCCATCGGTTTCC
Gm13_33031727_T_C	ACGTTGGATGCAATGGAGCTTATTAAGAAGG	ACGTTGGATGTTCCCTTCACATCCTTGGAG	GCTTATTAAGAAGGTATGCCAG
Gm13_33302559_G_A	ACGTTGGATGAACGCGGTGGTAATCATTGG	ACGTTGGATGAGGGTTCACTCCCAGACCTT	gggagTGTGCCACGTGGTAAAAAG
Gm13_33471044_G_A	ACGTTGGATGCAACAAGTGATCTCGACTCC	ACGTTGGATGTGACACAAGAAAGAAAATC	ggtgACGAGTCAAAGCTAAAGAATAA
Gm13_34624477_T_C	ACGTTGGATGTTGAGCAGCGGCTAAAAGAG	ACGTTGGATGAGACAGCTTTTATCCCATCC	gtgatCAGCGGCTAAAAGAGAGGCCA
Gm13_34778592_C_T	ACGTTGGATGCGTACGTAATTTTAGGAGGC	ACGTTGGATGATTGCTGCTTTCATTATCCC	ggatGAGGCGCGTTTTTATCGACTG
Gm13_35053712_C_A	ACGTTGGATGTCAGGAATTGGTAGGTCAGG	ACGTTGGATGCTCTGCCATTACTGCCAAAG	gGGTCAGGCACAGTAAC
Gm13_35188028_A_G	ACGTTGGATGAACACAACCGCTTTTCTCAC	ACGTTGGATGTGAAGGGCTTATGGAGAGTC	ctcgTTTCTCACAAGCATCCTCTG
Gm13_35492939_T_C	ACGTTGGATGCAAAACCTAACCGCACACTG	ACGTTGGATGTTTGTTGCCGCTCTCATCAC	tcccGCTACGCACAACAAAAATAACCCA
Gm13_36007185_T_C	ACGTTGGATGCCTTGGCCAAACTTATCCTC	ACGTTGGATGGAAAGTTGTCGATGGTGGTG	ccctAGTCAACTCAACCTCAC
Gm13_36188290_G_A	ACGTTGGATGGAGACACGTAGATGGAACAC	ACGTTGGATGAGTGTGTGAGGGAAGATTCG	ccctATGGAACACATTAACACAGCGAG
Gm13_36252057_G_A	ACGTTGGATGATACAATTGAAAGCAGGGAC	ACGTTGGATGGCAGATGGTTGGTATGAAAAG	cGGGAGAAAGGGTATGG
Gm13_36445455_T_C	ACGTTGGATGACTCTTCCCTCATGCTGATG	ACGTTGGATGACTTGTCTCCTTACACCCAC	ACCTTTAAGCTCATAGAGCAT
Gm13_38030960_T_C	ACGTTGGATGCATCAGCATACCTTGAACAG	ACGTTGGATGCCTGTTTGCACATGTTCCAT	ACAGAAAGACAAAGGTTATG
