# Supplementary material for: Identical Substitutions in Magnesium Chelatase Paralogs Result in Chlorophyll-Deficient Soybean Mutants
Source: G3 (Bethesda). 2014 Dec 1;5(1):123–31. doi: 10.1534/g3.114.015255 (PMC4291463; doi:10.1534/g3.114.015255)
Supplement: Supporting Information [file supp_g3.114.015255_TableS2.pdf]

Table S2 Primer sequences for Sequenom MassARRAY assays used for the second round of fine-mapping for the MinnGold mutation.

SNP_ID	2nd-PCRP	1st-PCRP	UEP_SEQ
Gm13_32186965	ACGTTGGATGACTGACTTTTTTCCCTTTCG	ACGTTGGATGCAGTGACGATAATTTTAAAGC	actagTTATACTCCCTGTCTCATTATGT
Gm13_32293065	ACGTTGGATGGAGTCTATCCGTTTTATCAC	ACGTTGGATGCGAGTTGCACGTACGAATAC	gtctcTCCGTTTTATCACTTTTACTC
Gm13_32316109	ACGTTGGATGAGGTGTACCTAACAATGCCC	ACGTTGGATGGTAGGTTAGCTAGTGTGTTG	cccaTCACAAGGCTTGGCGTCA
Gm13_32331620	ACGTTGGATGAGAAACATCCGTATGGTTCA	ACGTTGGATGTTTTTGGACTCCGTATGGAC	cccacTCCGTATGGTTCATACAAATT
Gm13_32343536	ACGTTGGATGTTTTAATATTAAGAGATTGG	ACGTTGGATGTATGACAACAGATTTGGTCC	TTTAATATTAAGAGATTGGATTAAGG
Gm13_32365734	ACGTTGGATGGGTGCTTATTTGAGGTTGAG	ACGTTGGATGGATACATAAAGTGAGTCTCT	tccaATATAACTTATTATTTGGCTGGT
Gm13_32586550	ACGTTGGATGGTGGGAGAGTTTTCATATAG	ACGTTGGATGCAAGCTAAGGACTGGTTGAC	cTTAGGAGTTGATTAGCGA
Gm13_32636705	ACGTTGGATGCACTAGGGCAGTGACTTCAT	ACGTTGGATGGGAATGTGCAAGTGTCTCTG	ctTCGCGTGCCTTTTTT
Gm13_32657362	ACGTTGGATGACGGATCACATTGGCTATGG	ACGTTGGATGGTTTCATTCGTGCATGCGAT	CATTGGCTATGGAATTCGATTA
Gm13_32673914	ACGTTGGATGGGTGAATTTGACGGGTGAAC	ACGTTGGATGATTCAGTTTGGGCTTGTCGC	GGTGAACTCAGTGGAA
Gm13_32954871	ACGTTGGATGTCACAGTAGGAGGTGTAGTC	ACGTTGGATGTAAAGTTGCTGCTCCAGCTC	aagatGAGGTGTAGTCGTAGGA
Gm13_32998715	ACGTTGGATGTTTTTCAAGATTGCGCTGGG	ACGTTGGATGAATAGAACCTTCACGGCTTC	ccgcTTGCGCTGGGTTGTGGT
Gm13_33026763	ACGTTGGATGATGCGCTTGAGTGGATGAAC	ACGTTGGATGTCTTCCGGAACTGCTTGTAG	TTGTTGGAAGAGAAAAAGCA
Gm13_33027247	ACGTTGGATGCTCCAGCCAAAGATTCTTCC	ACGTTGGATGCTAGGGTCCAAAATTCTCTG	TAGCCAACTCTATTGCAATAATGG
Gm13_33031727	ACGTTGGATGCAATGGAGCTTATTAAGAAGG	ACGTTGGATGTTCCCTTCACATCCTTGGAG	CTTATTAAGAAGGTATGCCAG
Gm13_33057712	ACGTTGGATGGCTTTATAGATGTTAGCCTTC	ACGTTGGATGGAGGAGAAGGTTGTTTGGTC	ctggTAGCCTTCATCTAAATGTCC
Gm13_33060476	ACGTTGGATGGACGGTTGTTCTTTTGTGGC	ACGTTGGATGCAGTTTCGTAATTACATTG	ccctcTCTTTTGTGGCTAATAAAAACTT
Gm13_33061634	ACGTTGGATGTCACAACATAACGTTGCAGG	ACGTTGGATGTCTAGCGCCACATAGTGAAG	ACGTTGCAGGTACAAAAA
Gm13_33063980	ACGTTGGATGCCTTCTTCGACTCTGCATTC	ACGTTGGATGCCCTCGGAAGTTGTAAAATC	aTCTGCATTCTTCTTCCT
Gm13_33124381	ACGTTGGATGACCATGGAGTCTGCAAGAAC	ACGTTGGATGGGGAGTTTGTCATGAGGATG	AGCAGCCCACACATCC
Gm13_33128103	ACGTTGGATGACATTGATGATTAGGGTGAC	ACGTTGGATGCAATTTACAACTTGTTCGGG	TGTCTAATTATATAAATACATTAGCTG
Gm13_33141206	ACGTTGGATGACAAACTCACAGCAGGAGTC	ACGTTGGATGTGTTGTGTTCTGCGCTTTGG	TATGTCTCTGGGTTTGGTG
Gm13_33146523	ACGTTGGATGCCAACTTGAGCATGCATTCC	ACGTTGGATGTCCTAATTGGCTCAGGCAAC	TCTAGCAGCTGCGGC
Gm13_33151333	ACGTTGGATGGTCACATAAATTGTACTAC	ACGTTGGATGGCTTTCCACTTACTCTGGTC	acAATCTTTAAGCAAACATCAAAT
Gm13_33191293	ACGTTGGATGACATTTGGCTTCCCTAGGTG	ACGTTGGATGCACACTCGCTCAGCGTGAT	aaacCCCTAGGTGGCTTCTTC
Gm13_33206265	ACGTTGGATGGCCTCATTTGGTCAGATATG	ACGTTGGATGGTCCTTATAAAACAACCAGCC	CTTTATTTGCTTATCATCCCTTT
Gm13_33212151	ACGTTGGATGGGAGCTGAAAGGTCTAAGAG	ACGTTGGATGAAAAAAATGCGCTCTCCTCCC	GTCTAAGAGCCTTTAAAACA
Gm13_33251230	ACGTTGGATGAGGTGCGCTTAGCGTAAATC	ACGTTGGATGAAAATTCACGCGATCTCACG	GCTAAACCCAGGAGG
Gm13_33271690	ACGTTGGATGTAAACTCTCTGCCCCTTTCC	ACGTTGGATGAGAAAGCAACTTGAGAGGCG	aagaTATTCTTTCTCTCCACGCTCTA
Gm13_33306556	ACGTTGGATGCTTCTACCAACCTTGATACC	ACGTTGGATGGAAATCCATGAGGGATGTTG	CAACCTTGATACCGCTAAC
Gm13_33309287	ACGTTGGATGTTACTATAAGTTAGTTTGTG	ACGTTGGATGTGCCCTTAAGGTGTTATCCC	TTACTATAAGTTAGTTTGTGTGATTAA
Gm13_33309683	ACGTTGGATGGCTTCAGCTCGTCAAGGAAC	ACGTTGGATGTCAGAATGAAACTCTTGCCC	CGTGGTCTCTGTGTG
Gm13_33429985	ACGTTGGATGCACTCGAATAAAGATCTCGG	ACGTTGGATGTATATGGGCGAGCCAAATTC	AGATCTCGGTTCTAAATCATTG
Gm13_33433989	ACGTTGGATGTTGCTTCTATGACAAACAC	ACGTTGGATGCAGAAAAGAAAAAACAGCCC	ACAAACACAATATTTACTACTTTTTAAT
Gm13_33434371	ACGTTGGATGCCATCATACTCTCACACAGG	ACGTTGGATGAGGTCTTGTTCCTCCTCTTC	ggaagCAGGAAACATTGAGACTC
Gm13_33439666	ACGTTGGATGGGACTAGAAATTAAGCTGTC	ACGTTGGATGGAAGAAATATCACTTTGGAAC	CATTTGTACACACTTGCCT
Gm13_33457770	ACGTTGGATGTTCACGAGTAGGGCAACAAC	ACGTTGGATGAAGAACGTCGTTGCCTATGG	TTCGGGTTACTTCGACTTAG
Gm13_33488403	ACGTTGGATGCCAACATCAATCATCCACCG	ACGTTGGATGATGGGTGGGCTTTAGATGAG	ACCGTCCGATCCTTC
Gm13_33505944	ACGTTGGATGTCACCACACCTTTTCCAACG	ACGTTGGATGGAAAGGTCATTGGAGTTGCG	tttcCAACTTTTCCGGCCATC

Gm13_33563545	ACGTTGGATGCCAAAACTCAAACAGAATG	ACGTTGGATGTGTGTTCATAATTTACCTC	tgGAACTAAAAATCAAGTGTTTAGTA
Gm13_33634870	ACGTTGGATGACAACAAGGTTCTGACCTCG	ACGTTGGATGGTGACCAATGACTTGTAGTG	tcgaCAACATGACCTGCGTTTTGAGC
Gm13_34213468	ACGTTGGATGGTCTTGAAACTTCTCCTCGG	ACGTTGGATGAAGAGGTTCAAGTGCTCCAG	cAGCCAAGCCTACGAC
Gm13_34233496	ACGTTGGATGTGTTAAGTGCAGGAACACTC	ACGTTGGATGTTGAATCCTCGTTTAGCCCG	cggggTAAGTGCAGGAACACTCAACTCT
Gm13_34248212	ACGTTGGATGACTCCTCCACGTGTTGAATC	ACGTTGGATGCGAGAACGGCTTTTACTTGG	gaaaGTGTTGAATCCATGCCCGC
Gm13_34293335	ACGTTGGATGAAAGGCCCATTTCGTGTTAG	ACGTTGGATGAGATCGGGTGGGTTAATTCG	attaTTTTTAATTTTAAAAAGGGCATAG
Gm13_34453742	ACGTTGGATGTCGCTATGTGTCACTTGTGG	ACGTTGGATGAGTTGTCAAGCTGCAATGCG	tcctAGCTCAACAACCTCTG
Gm13_34465720	ACGTTGGATGTTCGCGATGGGTTTCTTCAG	ACGTTGGATGTCTGATCCGGGAATGTTGTC	GCTTCGACAACTCCTC
Gm13_34602307	ACGTTGGATGTTGTTATGGGCCAAAAGTGC	ACGTTGGATGGCCTTATTTCATTACTGTGG	GGGCCAAAAGTGCCAAATTATATC
Gm13_34645498	ACGTTGGATGTTTTAAAAGGGTCGAGTCGG	ACGTTGGATGTGATCCGATTAAATCCGGTC	cccctTCGGACCGGTCTAACCGGGATC
Gm13_34701345	ACGTTGGATGCTTGTGATTTCTGTCTCCTC	ACGTTGGATGTTTGGCCACTTCTTCACCTC	tctaCTCATTTCTCATTTTCATGTG
Gm13_34748233	ACGTTGGATGATTGTCAAATGCACCCGAGC	ACGTTGGATGCTGTGGTGCATCCAATTGTG	AGCTCGATTCATCAAAACTTC
Gm13_34759965	ACGTTGGATGGAATCATCATTCATGTTCG	ACGTTGGATGCATTCTCTCTGTGCAATCCA	ccctAATCATCATTCATGTTCGTTTTAG
Gm13_34778592	ACGTTGGATGCGTACGTAATTTTAGGAGGC	ACGTTGGATGATTGCTGCTTTCATTATCCC	ccccaCGCGTTTTTATCGACTG
Gm13_34801784	ACGTTGGATGCGGGACGGAACTAGAAAAAA	ACGTTGGATGCCTCCAAAGACACTAAAAAG	AAAGAAAAAAATATTGCTTAACTTAT
Gm13_34808743	ACGTTGGATGTCTGTATCCGTGTCGTGTCC	ACGTTGGATGAGAGTTCAGACAATGTGAAG	gggcTCCGGTGTCCATTTCG
Gm13_34810502	ACGTTGGATGAGTTGGATGGCATCCTGAAC	ACGTTGGATGCTACACCATGAGCAGTTGGC	TGATCTATTACTTATTTATGGATAAAGA
Gm13_34819160	ACGTTGGATGTGTCTGCTACTTTTTCCCCC	ACGTTGGATGCTTGTTTCGGGTCACTAGTT	TCCCCCATATTTTGGATA
Gm13_34826532	ACGTTGGATGGCCTCAACGGACATCCAAAG	ACGTTGGATGCCTTTGCGGAATTAGATGGC	ACATCCAAAGAAACCGT
Gm13_34828053	ACGTTGGATGGCTCGAATACATGGACGAGG	ACGTTGGATGGCCTATCATATCCATCTGCG	GGACGAGGTATGCTCAT
Gm13_34831302	ACGTTGGATGAGTACTTAACATCTCCTAC	ACGTTGGATGTTTGTTTGAGGCTGTTTGAC	TTAACATCTCCTACTATCACT
Gm13_34855829	ACGTTGGATGATGTCAGGTGGTTCTAGTGG	ACGTTGGATGACCTCCTCATATTCTGCCTC	tTTCTCAGGGTGGGGA
Gm13_34859558	ACGTTGGATGAGTTATTTGGGGTCTGGTGG	ACGTTGGATGATGCACCTCCAAGGAGAAAC	ggaagGGTGGGACCAAAGTGTAT
Gm13_34861415	ACGTTGGATGTGACCAAGGTGTTTGACCTG	ACGTTGGATGGCCATATCTAGGACTAAAATC	aTCTCAAGTCTCAAGCTAA
Gm13_34862245	ACGTTGGATGGAGAACAACAACAAAGCTAC	ACGTTGGATGGTTACTGTCATAGTGCCCTG	caaaAACAAAGCTACAACAAAGTAA
Gm13_34870402	ACGTTGGATGTTCTGGATCGTGTTCAGGAG	ACGTTGGATGAACCCAGCTTTTAGTTTCCC	CGTGTTCAGGAGGAAATT
Gm13_34871976	ACGTTGGATGCTGAGTTAGGTAGGAATAGG	ACGTTGGATGGACCAACAGTTCAAAACGTC	AGGTAGGAATAGGATCACTTGA
Gm13_34882559	ACGTTGGATGGCCCACTAAATTTGTGGCTA	ACGTTGGATGATGTTGAACGTAATCCTCTC	CAATGTATTGTGAATTGTAATATTATTG
Gm13_34888379	ACGTTGGATGCGCCCTTTCTGTTAAGTGTG	ACGTTGGATGTCTATCCATTCGACACGTGC	ggggtTGTGATTGTGAAAGAATCC
Gm13_34890355	ACGTTGGATGATTATGGTGGTCTCACTCGC	ACGTTGGATGGTCTTACCGTAATTTTGGA	cgagaTGTGCATTGGTAGCATTGCG
Gm13_34895902	ACGTTGGATGTGATCTCAAGGGCATTGGAC	ACGTTGGATGTCCGAGCTTCTCTGACTGTG	gGGAGGTAGCGAACTG
