# Supplementary material for: Identical Substitutions in Magnesium Chelatase Paralogs Result in Chlorophyll-Deficient Soybean Mutants
Source: G3 (Bethesda). 2014 Dec 1;5(1):123–31. doi: 10.1534/g3.114.015255 (PMC4291463; doi:10.1534/g3.114.015255)
Supplement: Supporting Information [file supp_g3.114.015255_TableS3.pdf]

Table S3 PCR Primers used to amplify Glyma13g30560 for *y11* and *y11-2*.

PCR Reaction	Type	Primer Pair First Reaction	Type	Primer Pair Second Reaction (Nested)
1	Forward	TGGCACCCACTAACATTTCC	Forward	TGGCACCCACTAACATTTCC
1	Reverse	CCAGTATCCTTTTTATTTAGGAGACC	Reverse	CCAGTATCCTTTTTATTTAGGAGACC
2	Forward	TGGCACCCACTAACATTTCC	Forward	CGTTTTTGTCTTAAAAGCTTGATT
2	Reverse	CACACAACACACAAAAGAATGG	Reverse	GTTTGCAGCACACCATCC
3	Forward	TGGCACCCACTAACATTTCC	Forward	TCTTCTTCTTCCAAGCCTTCC
3	Reverse	CACACAACACACAAAAGAATGG	Reverse	CACACAACACACAAAAGAATGG
4	Forward	GGCCAGGCCTTTGCATTTTG	Forward	GGCCAGGCCTTTGCATTTTG
4	Reverse	ACTCAGCACACACCTTGGAG	Reverse	ACTCAGCACACACCTTGGAG
5	Forward	CCTGACTGAGGGTGTCAAGG	Forward	CCTGACTGAGGGTGTCAAGG
5	Reverse	GAAGTTAATCTTGGAGTGTATTTTGC	Reverse	GGCACTTACGTTGTCTCTTCC
6	Forward	CCTGACTGAGGGTGTCAAGG	Forward	GCTGAGTTGAATGTGGATGG
6	Reverse	GAAGTTAATCTTGGAGTGTATTTTGC	Reverse	GAAGTTAATCTTGGAGTGTATTTTGC
