# Supplementary material for: Identical Substitutions in Magnesium Chelatase Paralogs Result in Chlorophyll-Deficient Soybean Mutants
Source: G3 (Bethesda). 2014 Dec 1;5(1):123–31. doi: 10.1534/g3.114.015255 (PMC4291463; doi:10.1534/g3.114.015255)
Supplement: Supporting Information [file supp_g3.114.015255_TableS4.pdf]

Table S4 PCR Primers used to amplify Glyma15g08680 for CD-5.

PCR Reaction	Type	Primer Pair First Reaction	Type	Primer Pair Second Reaction (Nested)
1	Forward	CGGAGACTGGTAAATGTGAGC	Forward	CGGAGACTGGTAAATGTGAGC
1	Reverse	CAGCACACCTCCAAAACAAG	Reverse	CAGCACACCTCCAAAACAAG
2	Forward	GGCTAGGCCTTTGTGTTTGA	Forward	GGCTAGGCCTTTGTGTTTGA
2	Reverse	AACGGGAAATGCTGATTGAG	Reverse	AACGGGAAATGCTGATTGAG
3	Forward	GCTGCTGGATAGGTTTGGAA	Forward	GCTGCTGGATAGGTTTGGAA
3	Reverse	AACGGGAAATGCTGATTGAG	Reverse	AACGGGAAATGCTGATTGAG
4	Forward	TCAATTGCGGTTCTTCCTTC	Forward	ACGTCAGCTGCTATGAATGG
4	Reverse	TGGCCTCTGGCTTTCTTTAG	Reverse	GTAGCTCCCAATGGCAAATC
5	Forward	TCAATTGCGGTTCTTCCTTC	Forward	TCAATTGCGGTTCTTCCTTC
5	Reverse	TGGCCTCTGGCTTTCTTTAG	Reverse	TGGCCTCTGGCTTTCTTTAG
