# Supplementary material for: Identical Substitutions in Magnesium Chelatase Paralogs Result in Chlorophyll-Deficient Soybean Mutants
Source: G3 (Bethesda). 2014 Dec 1;5(1):123–31. doi: 10.1534/g3.114.015255 (PMC4291463; doi:10.1534/g3.114.015255)
Supplement: Supporting Information [file supp_g3.114.015255_TableS5.pdf]

Table S5 Twenty-two genes models present in the fine-mapped interval Gm13: 33,141,206..33,306,556

Gene	Location	Gene Annotation
Glyma13g30550	Gm13: 33141000..33143927	Transferase family
Glyma13g30560	Gm13: 33144571..33147208	Magnesium chelatase, subunit ChlI
Glyma13g30575	Gm13: 33147978..33151645	Peroxisomal NUDIX hydrolase
Glyma13g30590	Gm13: 33154582..33158861	Family not named
Glyma13g30600	Gm13: 33163957..33165210	Zinc finger, C3HC4 type (RING finger)
Glyma13g30610	Gm13: 33171016..33182189	ATP-Dependent RNA Helicase
Glyma13g30620	Gm13: 33183067..33188556	Glutamate-gated kainate-type ion channel receptor subunit GluR5 and related subunits
Glyma13g30625	Gm13: 33193010..33194243	There are no functional annotations for this locus
Glyma13g30630	Gm13: 33193058..33193890	There are no functional annotations for this locus
Glyma13g30650	Gm13: 33194700..33195205	There are no functional annotations for this locus
Glyma13g30640	Gm13: 33194530..33201169	Glutamate-gated kainate-type ion channel receptor subunit GluR5 and related subunits
Glyma13g30660	Gm13: 33204225..33209549	Glutamate-gated kainate-type ion channel receptor subunit GluR5 and related subunits
Glyma13g30670	Gm13: 33210247..33214795	D-Tyr-tRNA (Tyr) deacylase
Glyma13g30680	Gm13: 33221124..33226612	GDSL-like Lipase/Acylhydrolase
Glyma13g30690	Gm13: 33231142..33237718	GDSL-like Lipase/Acylhydrolase
Glyma13g30710	Gm13: 33243653..33244787	Regulation of transcription, DNA-dependent
Glyma13g30720	Gm13: 33247690..33248688	Regulation of transcription, DNA-dependent
Glyma13g30730	Gm13: 33259622..33263250	Pterin carbinolamine dehydratase PCBD/dimerization cofactor of HNF1
Glyma13g30740	Gm13: 33263320..33264787	Protein of unknown function, DUF599
Glyma13g30750	Gm13: 33280224..33286703	Auxin response factor
Glyma13g30760	Gm13: 33291706..33297139	Mlo family
Glyma13g30770	Gm13: 33302261..33303185	Glutaredoxin
