# Supplementary material for: Identical Substitutions in Magnesium Chelatase Paralogs Result in Chlorophyll-Deficient Soybean Mutants
Source: G3 (Bethesda). 2014 Dec 1;5(1):123–31. doi: 10.1534/g3.114.015255 (PMC4291463; doi:10.1534/g3.114.015255)
Supplement: Supporting Information [file supp_g3.114.015255_TableS6.pdf]

Table S6 Soybean CHLI Genes and Expression Data. Normalized soybean expression data showing that Glyma13g30560 and Glyma15g08680 share similar expression patterns, however Glyma13g30560 showing approximately twice the expression level in leaf tissues. Source: Severin, AJ, Woody JL, Bolon YT, Joseph B, Diers BW, et al. (2010) RNA-Seq Atlas of Glycine max: a guide to the soybean transcriptome. BMC Plant Biol 10:160.

Gene	Young leaf	Flower	One cm pod	Pod shell 10DAF	Pod shell 14DAF	Seed 10DAF	Seed 14DAF	Seed 21DAF	Seed 25DAF	Seed 28DAF	Seed 35DAF	Seed 42DAF	Root	Nodule
Glyma13g30560	106	16	38	41	33	2	3	6	8	6	8	3	1	1
Glyma15g08680	51	8	16	18	17	2	5	7	9	5	7	3	1	1
Glyma07g32550	2	3	2	1	1	1	1	1	1	0	0	0	2	1
Glyma13g24050	0	2	0	0	1	1	2	0	1	0	1	0	1	1
