# Supplementary material for: Identical Substitutions in Magnesium Chelatase Paralogs Result in Chlorophyll-Deficient Soybean Mutants
Source: G3 (Bethesda). 2014 Dec 1;5(1):123–31. doi: 10.1534/g3.114.015255 (PMC4291463; doi:10.1534/g3.114.015255)
Supplement: Supporting Information [file supp_g3.114.015255_FigureS1.pdf]

Figure S1

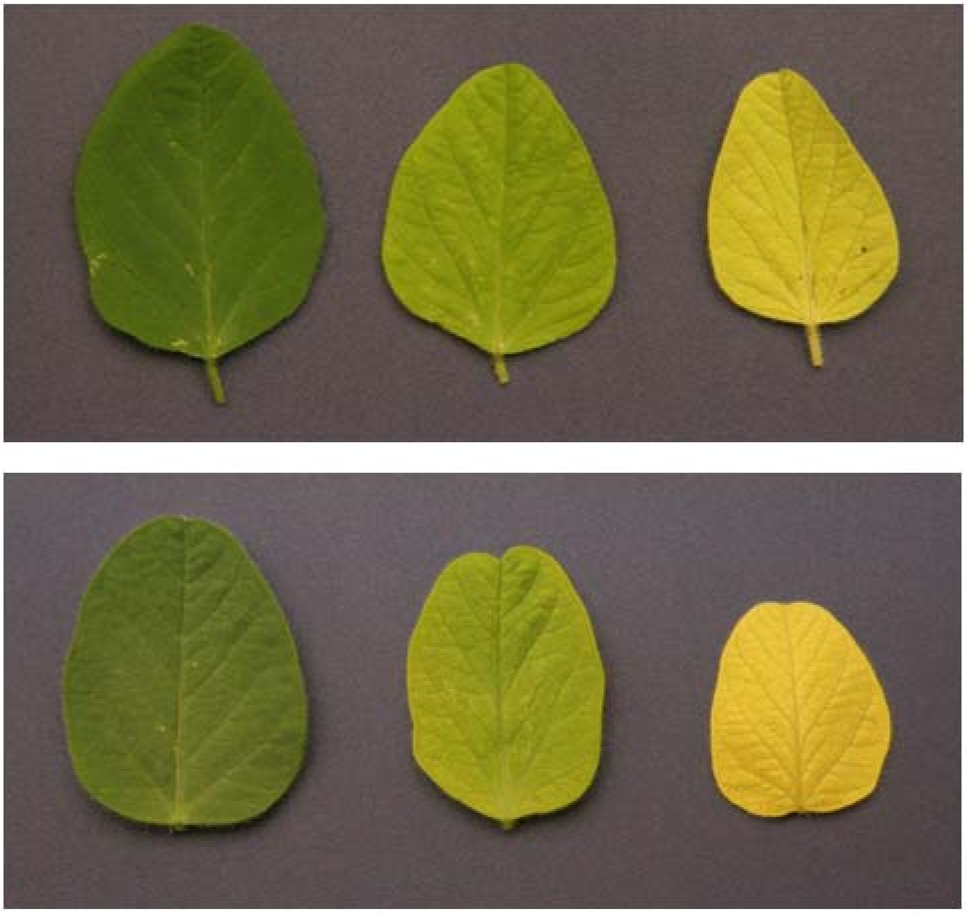

Figure S1 Phenotypic classes for chlorophyll deficiency mutants. (A) Leaves of *Y11/Y11*, *Y11/y11*, and *y11/y11* leaves showing the distinctive phenotypic classes. (B) Leaves of WT/WT, WT/CD-5, and CD-5/CD-5 leaves showing the distinctive phenotypic classes.
