# Supplementary material for: Identical Substitutions in Magnesium Chelatase Paralogs Result in Chlorophyll-Deficient Soybean Mutants
Source: G3 (Bethesda). 2014 Dec 1;5(1):123–31. doi: 10.1534/g3.114.015255 (PMC4291463; doi:10.1534/g3.114.015255)
Supplement: Supporting Information [file supp_g3.114.015255_FigureS3.pdf]

Figure S3

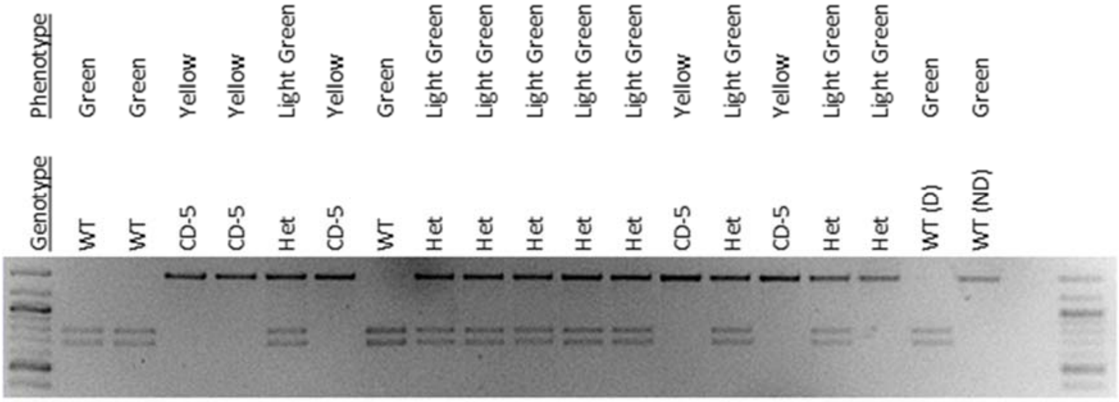

Figure S3 A Cleaved Amplified Polymorphic Sequences (CAPS) assay of seventeen individuals segregating for the presence of the candidate CD-5 SNP. In the rightmost lanes, D indicates a WT/WT digested sample and a ND indicates a WT/WT sample that was not digested. The perfect cosegregation of the candidate CD-5 SNP with the foliage phenotype provides additional information to suggest that the candidate SNP is responsible for the chlorophyll deficient phenotype.
