# Supplementary material for: Identical Substitutions in Magnesium Chelatase Paralogs Result in Chlorophyll-Deficient Soybean Mutants
Source: G3 (Bethesda). 2014 Dec 1;5(1):123–31. doi: 10.1534/g3.114.015255 (PMC4291463; doi:10.1534/g3.114.015255)
Supplement: Supporting Information [file supp_g3.114.015255_FigureS4.pdf]

Figure S4

A

```
                     Gm15:6,137,439...6137419...6,137,410
Consensus            AGAAGGTGAGCTCCGGCCACAGCTGCTGGA
CD-5                 ....................G.........
Wm82                 ....................A.........
Archer               ....................A.........
Minsoy               ....................A.........
Noir1                ....................A.........
Forrest              ....................A.........
Hendricks            ....................A.........
Missoy               ....................A.........
Parker               ....................A.........
Bert                 ....................A.........
Anderson             ....................A.........
Dunfield             ....................A.........
Lincoln              ....................A.........
Odgen                ....................A.........
Perry                ....................A.........
Richland             ....................A.........
                                          CAG Glutamine
                                          CGG Arginine
```

B

```
Glyma13g30560            AGAAGGGGAGCTCCGGCCGCAGCTGCTAGA
Glyma15g08680            AGAAGGTGAGCTCCGGCCACAGCTGCTGGA
SNP differences          ......*...........*........*..

Glyma13g30560 y11        ......G...........G.G......A..
Glyma13g30560 y11-2      ......G.......A...G........A..

Glyma15g08680 CD-5       ......T...........A.G......G..
```

Figure S4 The CD-5 mutation in the candidate gene Glyma15g08680 appears to be a novel *de novo* mutation. (A) Sequence results of CD-5 compared to a panel of diverse soybean lines. Of the 29 diverse soybean lines sequenced (15 are shown), only CD-5 was found to have Guanine rather than Adenine at position 6137419. The CD-5 SNP causes a nonsynonymous change from Glutamine to Arginine. (B) Sequences from Glyma13g30560 and Glyma15g08680 showing that the *y11* and CD-5 SNPs in the separate genes occur at the same relative SNP position, but that sequences from the two genes can be differentiated by nearby SNPs. Sequences for Glyma13g30560 and Glyma15g08680 are from positions Gm13:33,146,509..33,146,538 and Gm15:6,137,439..6,137,410, respectively.
