# Supplementary material for: Identical Substitutions in Magnesium Chelatase Paralogs Result in Chlorophyll-Deficient Soybean Mutants
Source: G3 (Bethesda). 2014 Dec 1;5(1):123–31. doi: 10.1534/g3.114.015255 (PMC4291463; doi:10.1534/g3.114.015255)
Supplement: Supporting Information [file supp_g3.114.015255_FigureS5.pdf]

Figure S5

```
Consensus  MAS.LGTSSI AVLPSR..SS .SSKPSIHTL SLTSGQ.YGR KFYGGIGIHG 50
Gma ChlI1a ...A...... ......YF.. S......... ......N... ..........
Gma ChlI1b ...T...... ......CI.. F......... ......S... ..........

Consensus  IKGR.QLSV. NVATEVNSVE QAQSIASKES QRPVYPFSAI VGQDEMKLCL 100
Gma ChlI1a ....A....T .......... .......... .......... ..........
Gma ChlI1b ....S....A .......... .......... .......... ..........

Consensus  LLNVIDPKIG GVMIMGDRGT GKSTTVRSLV DLLPEIKVVA GDPYNSDPQD 150
Gma ChlI1a .......... .......... .......... .......... ..........
Gma ChlI1b .......... .......... .......... .......... ..........

Consensus  PEFMGVEVRE RVLQGEELSV VLTKINMVDL PLGATEDRVC GTIDIEKALT 200
Gma ChlI1a .......... .......... .......... .......... ..........
Gma ChlI1b .......... .......... .......... .......... ..........

Consensus  EGVKAFEPGL LAKANRGILY VDEVNLLDDH LVDVLLDSAA SGWNTVEREG 250
Gma ChlI1a .......... .......... .......... .......... ..........
Gma ChlI1b .......... .......... .......... .......... ..........

Consensus  ISISHPARFI LIGSGNPEEG ELRPQLLDRF GMHAQVGTVR DAELRVKIVE 300
Gma ChlI1a .......... .......... .......... .......... ..........
Gma ChlI1b .......... .......... .......... .......... ..........

Consensus  ERGRFDKNPK EFRDSYKAEQ EKLQQQITSA RSVLSSVQID QDLKVKISKV 350
Gma ChlI1a .......... .......... .......... .......... ..........
Gma ChlI1b .......... .......... .......... .......... ..........

Consensus  CAELNVDGLR GDIVTNRAAK ALAALK.RD. VSAEDIATVI PNCLRHRLRK 400
Gma ChlI1a .......... .......... ......G..N .......... ..........
Gma ChlI1b .......... .......... ......E..K .......... ..........

Consensus  DPLESIDSGL LVTEKFYEVF S 421
Gma ChlI1a .......... .......... .
Gma ChlI1b .......... .......... .
```

Figure S5 Amino acid sequence comparison of ChlI1a (Glyma13g30560) to ChlI1b (Glyma15g08680) showing the high degree of similarity between the two Mg-chelatase subunits. The two boxed residues indicate the positions of the *y11-2* (R273Q) mutation and *y11* and CD-5 (Q275R) mutations.
