# Supplementary material for: Identical Substitutions in Magnesium Chelatase Paralogs Result in Chlorophyll-Deficient Soybean Mutants
Source: G3 (Bethesda). 2014 Dec 1;5(1):123–31. doi: 10.1534/g3.114.015255 (PMC4291463; doi:10.1534/g3.114.015255)
Supplement: Supporting Information [file supp_g3.114.015255_FigureS6.pdf]

Figure S6

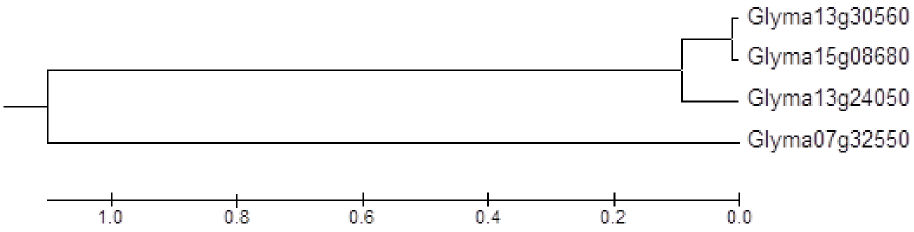

Figure S6 The inferred evolutionary history for the four Glycine Max ChlI subunits calculated using the UPGMA method. The UPGMA neighbor joining tree shows the close sequence similarity of Glyma13g30560 with Glyma15g08680. The optimal tree with the sum of branch length = 2.30757376 is shown.
